# Exploring the Anti-Inflammatory and Antioxidant Potential, Metabolite Composition and Inorganic Profile of *Cistus monspeliensis* L. Aerial Parts and Roots

**DOI:** 10.3390/antiox13070753

**Published:** 2024-06-21

**Authors:** Eileen Mac Sweeney, Ilaria Chiocchio, Manuela Mandrone, Cinzia Sanna, Fabjola Bilo, Giuseppina Maccarinelli, Vlad Sebastian Popescu, Mariachiara Pucci, Stefania Morandini, Maurizio Memo, Daniela Letizia Uberti, Laura Borgese, Simona Trincia, Ferruccio Poli, Andrea Mastinu, Giulia Abate

**Affiliations:** 1 Department of Molecular and Translational Medicine, Division of Pharmacology, University of Brescia, 25123 Brescia, Italy; e.macsweeney@studenti.unibs.it (E.M.S.); giuseppina.maccarinelli@unibs.it (G.M.); vlad.popescu@unibs.it (V.S.P.); mariachiara.pucci@unibs.it (M.P.); stefania.morandini@unibs.it (S.M.); maurizio.memo@unibs.it (M.M.); daniela.uberti@unibs.it (D.L.U.); giulia.abate@unibs.it (G.A.); 2 Department of Pharmacy and Biotechnology (FaBit), Alma Mater Studiorum, University of Bologna, Via Irnerio 42, 40126 Bologna, Italy; ilaria.chiocchio2@unibo.it (I.C.); simona.trincia2@unibo.it (S.T.); ferruccio.poli@unibo.it (F.P.); 3 Department of Life and Environmental Sciences, University of Cagliari, Via S. Ignazio da Laconi 13, 09123 Cagliari, Italy; cinziasanna@unica.it; 4 Department of Mechanical and Industrial Engineering, University of Brescia, Via Branze 38, 25123 Brescia, Italy; fabjola.bilo@unibs.it (F.B.); laura.borgese@unibs.it (L.B.)

**Keywords:** Cistaceae, RAW 264.7, antioxidant defense, mitochondria, TXRF, cytokines, ^1^H NMR profiling, methoxy flavonols, inorganic profile

## Abstract

This work focuses on *Cistus monspeliensis* L. aerial parts (AP) and roots (R) extracts, investigating the anti-inflammatory and antioxidant potential of the two organs in comparison. At dosages between 1.56 and 6.25 µg/mL, both extracts showed a protective effect against LPS inflammatory stimulus on a macrophage cell line (RAW 264.7). Interestingly, only R was able to significantly reduce both IL-1β and IL-6 mRNA gene expression in the presence of LPS. Moreover, the treatment of a neuroblastoma cell line (SH-SY5Y) with AP and R at 6.25 µg/mL increased the cell survival rate by nearly 20% after H_2_O_2_ insult. However, only R promoted mitochondria survival, exhibited a significantly higher production of ATP and a higher activity of the enzyme catalase than the control. Both AP and R had similar primary metabolites; in particular, they both contained 1-*O*-methyl-*epi*-inositol. Labdane and methoxylated flavonoids were the most characteristic compounds of AP, while R contained mainly catechins, gallic acid, and pyrogallol derivatives. Considering the importance of elemental composition in plants, the inorganic profile of AP and R was also investigated and compared. No potentially toxic elements, such as Pb, were detected in any sample.

## 1. Introduction

*Cistus* L. is a genus of dicotyledonous perennial plants with pink or white flowers and viscid stems [1,2,3]. It belongs to the rockrose family Cistaceae and includes 21 species [4]. The most common *Cistus* species in the Mediterranean region are *C. albidus* L., *C. creticus* L., *C. crispus* L., *C. parviflorus* Lam., *C. monspeliensis* L., *C. populifolius* L., *C. salviifolius* L., *C. ladanifer* L., *C. laurifolius* L., and *C. clusii* Dunal [1].

These plants can be found from the sea level to the mountainous regions, both in acidic and basic soils; they are resistant to summer droughts and able to grow in fire-degraded soils [1,4,5,6]. In particular, on Sardinia Island (Italy), *Cistus* is one of the most representative shrub genera, accounting for six taxa [2].

The glandular trichomes present on the leaves of these plants produce a characteristic resin named ladano, which is responsible for the aromatic scent and contains compounds of pharmacological interest [1,3,7]. Although each species has a distinct phytochemical profile, terpenoids (sesquiterpenes, diterpenes, monoterpenes), polyphenols (flavonoids, tannins, and phenolic acids), coumarins, and alkaloids are commonly found in the aerial parts of *Cistus* species [8,9,10,11]. Moreover, their essential oils contain mainly monoterpenes, sesquiterpenes, and diterpenes [8].

This work is focused on *Cistus monspeliensis* L. (Montpelier rockrose), a lowland shrub dominant in evergreen garrigue vegetation [1,4,5,6] belonging to the white-flowered *Cistus* lineage [1]. It is one of the most common *Cistus* species in the Mediterranean basin [7], where it is used as a traditional remedy to treat wounds [6]. In Sardinian folk medicine, the poultice obtained from fresh leaves is topically used for wound healing, skin diseases, and as an analgesic; additionally, the infusion is employed to treat tick bites [12,13,14].

Aerial parts of *C. monspeliensis* showed anti-inflammatory efficacy in vivo against paw edema [5], as well as antimicrobial activity against several bacteria and fungi strains [15,16,17]. Moreover, its antioxidant activity, determined through simple in vitro colorimetric tests (DPPH and BCB), was also explored in comparison with other *Cistus* species [18,19], and *C. monspeliensis* resulted the most active one. Attaguile et al. [19] found that in addition to showing antioxidant and superoxide in vitro scavenging activity, *C. monspeliensis* aqueous extract was also able to inhibit lipoperoxidation in rat liver microsomes. Regarding its essential oil, this resulted in antioxidant and enzyme inhibitory activity [3]. Despite the ethnobotanical relevance and these promising results obtained for *C. monspeliensis* aerial parts, to the best of our knowledge, there are no studies focused on its antioxidant and anti-inflammatory activity in cell models, deepening the mechanisms of action. Moreover, there are no previous studies on the roots of this plant, which are not mentioned in any ethnobotanical reports and still remain unknown in terms of both phytochemical profile and biological activity.

Hence, the present study aims to extend the knowledge of *C. monspeliensis*, explore its antioxidant and anti-inflammatory potential in cell models, and test both aerial parts and root extracts in comparison. An overview of the phytochemical profile of the two organs was acquired by ^1^H NMR profiling, followed by further phytochemical analysis. Moreover, to have a complete overview of this plant composition, the inorganic profile was also investigated, considering the importance of elementals for human health and safety and the fact, widely reported in the literature, that plant metal content can also influence its biological activity [20].

## 2. Materials and Methods

### 2.1. Reagents

All reagents used for cell cultures were purchased from Sigma Aldrich (Merck KGAA, Darmstadt, Germany), including Roswell Park Memorial Institute medium (RPMI 1640), Dulbecco’s Modified Eagle Medium (DMEM), Ham’s F12 medium, fetal bovine serum, L-glutamine, penicillin and streptomycin. For the cell treatments and antioxidant assays, dimethyl sulfoxide (DMSO), Phosphate Buffered Saline (PBS), Lipopolysaccharide (LPS), H_2_O_2_, MTT (3-[4,5-dimethylthiazol-2-yl]-2,5-diphenyl-tetrazolium bromide), Ammonium molybdate and purified Catalase enzyme (20100U) were obtained from Sigma Aldrich (Merck KGAA, Darmstadt, Germany). MitoTracker™ Green FM, for the mitochondrial content, was from Thermo Fisher Scientific (Waltham, MA, USA). To evaluate the ATP production, Seahorse XFe24 culture plates and Agilent Seahorse XF base medium DMEM were purchased from Agilent Technologies (Agilent, Santa Clara, CA, USA); poly-L-lysine, sodium pyruvate, Glucose, glutamine, HEPES, oligomycin, rotenone, and antimycin A were from Sigma Aldrich (Merck KGAA, Darmstadt, Germany). The Qubit protein assay organic dye was obtained from Invitrogen (Buenos Aires, Argentina). Regarding qPCR, TRIzol was from Sigma Aldrich (Merck KGAA, Darmstadt, Germany), while oligo (dT) primers, M-MLV reverse transcriptase, dNTPs, RNase inhibitor, and 1X RT buffer were obtained from Promega (Madison, WI, USA); SYBR Green Master Mix was from Bio Rad Laboratories (Richmond, CA, USA).

### 2.2. Plant Collection and Extraction

**Figure 1:**
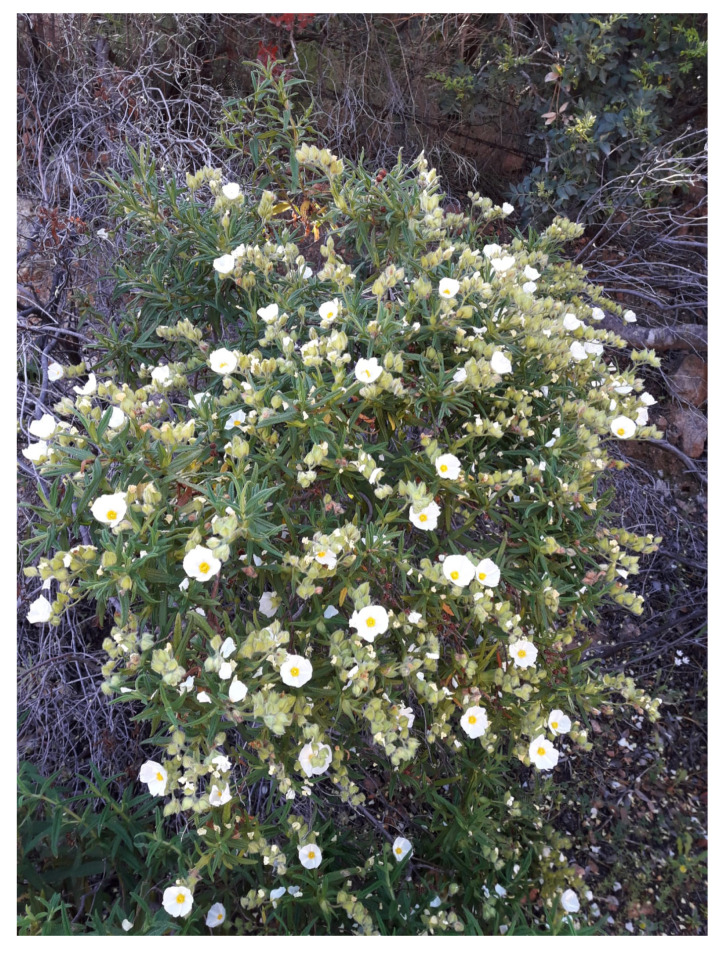
*Cistus monspeliensis* L.

Aerial parts (AP) and roots (R) from five plants of *C*. *monspeliensis* (Figure 1) were collected during the spring of 2016 from the Paringianu area, Sardinia, Italy (39°10′19.0″ N 8°27′31.9″ E). The plant is not threatened or endangered and it is not protected by national or international regulations. The whole plant was manually removed from the soil, and the aerial parts and roots were separated and carefully stored in zip-locked polyethylene bags with silica gel for transport. Subsequently, samples were dried at 40 °C in a ventilated oven and kept in sealed recipients in the dark. Subsequently, hydroalcoholic extraction and all the biological and chemical analyses were performed between 2017 and 2018. A voucher specimen (CAG 135v) was deposited in the Herbarium of the University of Cagliari (Italy).

### 2.3. Preparation of Plant Extracts for Bioactivity Tests and for NMR Profiling

For biological assays, plant material was extracted following the method proposed by Cappadone et al. [13] with slight modifications. Briefly, 120 mg of dried and powdered plant material were extracted by sonication for 20 min using 6 mL of MeOH/H_2_O (1:1). Subsequently, samples were centrifuged (1700× *g*) for 20 min, and the supernatant was separated from the pellet, aliquoted in tests tubes, and dried in vacuum concentrator (SpeedVac SPD 101b 230, Savant, Italy) yielding in total 38 mg of AP and 6 mg of R extracts. This procedure is optimized to obtain a wide range of metabolites with a minimal consumption of both solvents and plant material.

To obtain the fingerprinting of the phytochemical composition of the extract, firstly, ^1^H NMR was employed. An extraction procedure similar to that used for the bioassay was carried out, using 30 mg of sample and as solvents 0.5 mL of MeOH-*d_4_* and 0.5 mL phosphate buffer (90 mM, pH 6.0) in H_2_O-*d_2_* containing standard 0.01% trimethylsilylpropionic-2,2,3,3-*d_4_* acid sodium salt (TMSP). This time the supernatant was directly transferred to NMR tubes and subjected to ^1^H NMR profiling and HSQC NMR experiments.

### 2.4. Fractionation for Compounds Identification

With the aim of obtaining an adequate yield to carry out the structure elucidation of the main secondary metabolites, in this stage, a different extraction procedure was employed.

One hundred g of dried and grounded AP were extracted through a maceration for 72 h using 700 mL of CH_3_OH/H_2_O (70:30). The extraction procedure was repeated three times on the same plant material, and the extract was filtered and dried in rotary evaporator yielding 34.8 g of dried extract. This latter was then suspended in 350 mL of water to undergo liquid–liquid partition (four times with each solvent). After anhydrification, the fractions were dried in a rotary evaporator, obtaining hexane (APHEx = 1 g), chloroform (APCl = 5 g), ethyl acetate (APEt = 2.1 g), and water (APW = 20 g) fractions. 8-Hydroxylabdan-15-oic acid was partially purified from APCl, while flavonols were obtained from both APCl and APEt; both were further subjected to Medium-Pressure Liquid Chromatography (MPLC).

APCl was dissolved in 7 mL of chloroform and mixed with 5 g of silica gel (60 g Kieselgel^®^, Merck KGaA, Darmstadt, Germany), then the solvent was evaporated in rotavapor, and the fraction was injected by dry loading in MPLC system (Reveleris^®^, Büchi, Switzerland) connected to a silica column (80 g, 40 µm). The eluent consisted of an isocratic phase of CHCl_3_:EtOH (97:3), the flow rate was 20 mL/min, and the run length was 60 min. The fractions were collected by volume (15 mL in each tube) and were successively reduced to 11 fractions, unifying on the basis of Thin Layer Chromatography (TLC) profiles.

The TLC was carried out using silica gel slides with 254 nm fluorescence indicator and as mobile phase CHCl_3_:EtOH (97:3). Compound 8-hydroxylabdan-15-oic acid was directly crystallized from subfraction 8 in the same subfraction also eluted quercetin 3,4′-dimethylether and myricetin 3,4′,5′-trimethyl ether.

Analogously, APEt was dry-loaded in the MPLC instrument connected to the same column. The eluent consisted of an isocratic phase of CHCl_3_:MeOH (95:5), the flow rate was 35 mL/min, and the run length was 80 min. The fractions were collected by volume (25 mL in each tube) and successively reduced to sixteen fractions, unifying on the basis of the TLC profiles as previously described. Compound myricetin 3,7,4′,5′-tetramethyl ether was obtained from subfraction 1 (and also found in APCl subfraction 4). Gallic acid was obtained from subfraction 14, and catechin and 1-(2-hydroxy-6-methylphenyl) ethanone 2-*O*-*β*-hexoside were obtained from subfraction 15.

Two hundred grams of dried and grounded R were extracted through maceration of 72 h using 1400 mL of CH_3_OH/H_2_O (70:30). The extraction procedure was repeated four times on the same plant material, and the extract was filtered and freeze-dried (yield 12 g). The extract was suspended in 500 mL of water to undergo liquid–liquid partition. After anhydrification, the fractions were dried in a rotary evaporator, obtaining chloroform (RCl = 110.9 mg), ethyl acetate (Ret = 865 mg), and water (RW = 8.18 g) fractions. 1-*O*-methyl-*epi*-inositol was detected in RW and prepurified through a Solid Phase Extraction (SPE) followed by size exclusion chromatography. Briefly, 1.2 g of RW was solubilized in the minimum volume of water and loaded into the Supelco Discovery^®^ DPA-6S SPE Tube (Merck KGaA, Darmstadt, Germany), eluting first with methanol and then with water. The water eluate was dried in a rotary evaporator. Subsequently, 300 mg of it was then solubilized in the minimum volume of methanol and loaded into a chromatography column (900 mm × 16 mm) filled with 60 g of Sephadex (LH-20). This latter chromatography was carried out using methanol as an eluent with a flow rate of 0.6 mL/min. A total of 80 tubes were collected, and 1-*O*-methyl-*epi*-inositol was eluted in tubes 19–21 and was chemically characterized from tube 20.

### 2.5. NMR Measurement and Analysis

^1^H NMR spectra and inverse detected ^1^H-^13^C correlation experiments (HMBC, HSQC) were recorded at 25 °C on a Bruker Avance Neo Ascend 600 instrument equipped with the cryoprobe Prodigy. For ^1^H NMR profiling, the instrument was operating at a ^1^H NMR frequency of 600.13 MHz, and MeOH-*d_4_* was used as an internal lock. Each ^1^H NMR spectrum consisted of 16 scans (corresponding to 5 min) with a relaxation delay (RD) of 2 s and spectral width of 9595.8 Hz (corresponding to δ 16.0). A presaturation sequence (PRESAT) was used to suppress the residual water signal at δ 4.83. The spectra were manually phased and baseline corrected and calibrated to the internal standard trimethyl silyl propionic acid sodium salt (TMSP) at δ 0.0 using Mestrenova software (version 15.0, Mestrelab Research, Spain). The standard was also used to perform a semiquantitative analysis of 1-*O*-methyl-*epi*-inositol and gallic acid. The analysis of the extracts ^1^H NMR profiles was performed based on an in-house library and compared with the literature [21,22]. All the spectra were uploaded onto the Zenodo repository (https://doi.org/10.5281/zenodo.11281288).

### 2.6. Analytical Chromatography

UHPLC-MS analysis was run on a Waters ACQUITY ARC UHPLC/MS system consisting of a QDa mass spectrometer equipped with an electrospray ionization interface and a 2489 UV/Vis detector. The detected wavelengths (λ) were 225 nm and 260 nm. The analyses were performed on an XBridge BEH C18 column (10 mm × 2.1 mm i.d., particle size 2.5 μm) with an XBridge BEH C18 VanGuard Cartridge precolumn (5 mm × 2.1 mm i.d., particle size 1.8 µm). The mobile phases were H_2_O (0.1% formic acid) (A) and MeCN (0.1% formic acid) (B). Electrospray ionization in positive (QDa 1) and negative (QDa 2) modes was applied in the mass scan range of 50–1200 Da. REt was diluted to 1 mg/mL, and a volume of 4 μL was injected. The extract was eluted with an explorative gradient starting with 5% B and reaching 95% B in 15 min. The flow rate was 0.8 mL/min. This analysis allowed gallic acid, catechin, and gallocatechin to be detected (Figure S1).

Analogously, APEt subfractions 1 and 15 and APCl subfraction 8 were chromatographed following the same procedure. This led to the identification of myricetin 3,7,4′,5′-tetramethyl ether, catechin, 1-(2-hydroxy-6-methylphenyl) ethanone 2-*O*-*β*-hexoside, myricetin 3,4′,5′-trimethyl ether, and quercetin 3,4′-dimethyl ether.

Finally, to confirm the presence of the characterized compounds in AP and R extracts, two aliquots prepared for the biological assays (one AP and one R) were solubilized at 10 mg/mL and subjected to the same UHPLC-MS analysis (values reported in the next paragraph).

### 2.7. Compounds Identification

Below, the ^1^H and ^13^C NMR resonances of the characterized compounds are reported, together with the *m*/*z* values and retention times (RT) found in AP and R.

#### 2.7.1. 8-Hydroxylabdan-15-oic Acid (**1**)

^1^H NMR spectral data (600 MHz, DMSO) δ: 3.82 (1 H, s, OH), 2.22 (1 H, dd, J = 14.88, 5.87 Hz, H14a), 1.96 (1 H, dd, J = 14.88, 8.3 Hz, H14b), 1.78 (1 H, m, H13), 1.67 (1 H, dt, H7a), 1.55 (1 H, H1b), 1.52 (1 H, H6b), 1.44 (1 H, H12b), 1.35 (1 H, H11b), 1.34 (1H, dt, H7b), 1.32 (1 H, H3b), 1.20 (1 H, H6a), 1.14 (1 H, H11a), 1.13 (1 H, H12a), 1.10 (1 H, H3a), 0.98 (3 H, s, H17), 0.93 (1 H, t, H9), 0.88 (3 H, d, J = 6.66 Hz, H16), 0.87 (1 H, H1a), 0.84 (1 H, d, J = 2.4 Hz, H5), 0.83 (3 H, s, H18), 0.75 (3 H, s, H19), 0.73 (3 H, s, H20). ^13^C NMR, δ: 175.11 (C15), 72.67 (C8), 62.07 (C9), 56.37 (C5), 44.43 (C7), 42.11 (C3), 41.87 (C14), 40.72 (C12), 39.95 (C1), 39.57 (C10), 33.76 (C18), 33.54 (C4), 31.33 (C13), 24.18 (C17), 22.67 (C11), 21.75 (C19), 20.60 (C6), 20.06 (C16), 18.23 (C2), 15.66 (C20). *m*/*z* value in QDa 2 = 323 [M-H]^−^ found in AP at RT 9.48 min.

#### 2.7.2. Gallic Acid (**2**)

^1^H NMR spectral data (600 MHz, D_2_O), δ: 7.03 (2H, s, H1′, H6′) ^13^C NMR, δ: 172.03 (C7), 144.64 (C4), 137.18 (C3, C5), 123.30 (C1), 110.0 (C2, C6). *m*/*z* value in QDa 2 = 169 [M-H]^−^ found in R at RT 0.53 min and in AP at RT 0.58 min.

#### 2.7.3. Myricetin 3,7,4′,5′-Tetramethyl Ether (**3**)

^1^H NMR spectral data (600 MHz, DMSO) δ: 9.63 (1H, s, 3′OH), 7.38 (1H, d, J = 2.11 Hz, H2′), 7.22 (1H, d, J = 2.11 Hz, H6′), 6.76 (1H, d, J = 2.09 Hz, H8), 6.40 (1H, d, J = 2.09 Hz, H6), 3.88 (3H, s, 7 OCH_3_), 3.87 (3H, s, 5′ OCH_3_), 3.83 (3H, s, 3 OCH_3_), 3.78 (3H, s, 4′ OCH_3_). ^13^C NMR, δ: 178.55 (C4), 165.85 (C7), 164.50 (C5), 156.84 (C9), 155.83 (C2), 153.49 (C5′), 151.03 (C3′), 139.42 (C4′), 139.22 (C3), 110.50 (C2′), 105.84 (C10), 104.3 (C6′) 98.4 (C6), 92.9 (C8), 60.64 (4′ OCH_3_), 60.38 (3 OCH_3_), 56.63 (7 OCH_3_), 56.40 (5′ OCH_3_). *m*/*z* value in QDa 1 = 375 [M+H]^+^ found in AP at RT 7.49 min.

#### 2.7.4. Quercetin 3,4′-Dimethyl Ether (**4**)

^1^H NMR spectral data (600 MHz, DMSO) δ: 12.68 (1H, s, 5OH), 10.88 (1H, s, 7OH), 9.63 (1H, s, 3′OH), 7.65 (1H, d, J = 2.03 Hz, H2′), 7.58 (1H, dd, J = 8.43, J = 2.03 Hz, H6′), 6.97 (1H, d, J = 8.43 Hz, H5′), 6.48 (1H, d, J = 2.10 Hz, H8), 6.22 (1H, d, J = 2.10 Hz, H6), 3.86 (3H, s, 4′OCH_3_), 3.81 (3H, s, 3OCH_3_). ^13^C NMR, δ: 178.41 (C4), 164.44 (C7), 161.66 (C5), 155.79 (C2), 149.90 (C3′), 148.58 (C4′), 138.79 (C3), 122.62 (C2′), 121.24 (C1′), 116.30 (C5′), 112.60 (C6′), 104.64 (C10), 98.93 (C6), 94.25 (C8), 60.09 (3OCH_3_), 56.24 (4′OCH_3_). *m*/*z* value in QDa 2 = 329 [M-H]^−^ found in AP at RT 5.82 min.

#### 2.7.5. Myricetin 3, 4′,5′-Trimethyl Ether (**5**)

^1^H NMR spectral data (600 MHz, DMSO) δ: 12.60 (1H, s, 5OH), 10.88 (1H, s, 7OH), 9.92 (1H, s, 3′OH), 7.24 (1H, d, J = 2.10 Hz, H2′), 7.19 (1H, d, J = 2.10 Hz, H6′), 6.46 (1H, d, J = 2.10 Hz, H8), 6.21(1H, d, J = 2.10 Hz, H6), 3.85 (3H, s, 5′OCH_3_), 3.80 (3H, s, 3OCH_3_), 3.77 (3H, s, 4′OCH_3_). ^13^C NMR, δ: 178.78 (C4), 164.44 (C7), 161.66 (C5), 156.42 (C9), 155.54 (C2), 152.39 (C5′), 150.77 (C3′), 139.30 (C4′), 138.2 (C3), 125.27 (C1′), 110.36 (C2′), 104.84 (C10), 104.31 (C6′), 98.93 (C6), 94.25 (C8), 60.34 (4′OCH_3_), 60.09 (3 OCH_3_), 56.24 (5′ OCH_3_). *m*/*z* value in QDa 2 = 359 [M-H]^−^ found in AP at RT 6.02 min.

#### 2.7.6. 1-(2-Hydroxy-6-methylphenyl) Ethanone 2-O-β-Hexoside (**6**)

^1^H NMR spectral data (600 MHz, D_2_O), δ: 7.26 (1H, t, H4), 6.98 (1H, d, J = 8.37 Hz, H3), 6.94 (1H, d, J = 8.37 Hz, H5), 5.01 (1H, d, J = 7.71 Hz, H1′), 3.45 (1H, H2′), 3.50 (1H, H3′), 3.40 (1H, H4′), 3.39 (1H, H5′), 3.65 (1H, H6′a), 3.83 (1H, H6′b), 2.51 (3H, s, H9), 2.18 (3H, s, H7). ^13^C NMR, δ: 218.51 (C8), 152.56 (C2), 135.51 (C6), 131.43 (C1), 130.10 (C4), 125.29 (C5), 112.72 (C3), 100.35 (C1′), 76.10 (C3′), 75.75 (C5′), 72.78 (C2′), 69.32 (C4′), 60.82 (C6′) 31.96 (C9), 17.91 (C7). *m*/*z* value in QDa 1 = 335.12 [M+Na]^+^ found in AP at RT 3.40 min.

#### 2.7.7. Catechin (**7**)

^1^H NMR spectral data (600 MHz, D_2_O), δ: 6.83 (1H, d, J = 1.89 Hz, H6′), 6.82 (1H, d, J = 8.28 Hz, H3′), 6.72 (1H, dd, J = 1.89, J2 = 8.28 Hz, H2′), 5.93 (1H, H6), 5.83 (1H, H8), 4.54 (1H, d, J = 7.75 Hz, H2), 4.01 (1H, m, H3), 2.76 (1H, dd, J = 5.43, 15.85 Hz, H4a), 2.37 (1H, dd, J = 8.36, 15.85 Hz, H4b). ^13^C NMR, δ: 155.21 (C5), 154.75 (C9), 156.42 (C9), 144.29 (C5′), 143.93 (C4′), 130.17 (C1′), 119.96 (C2′), 116.19 (C3′), 114.94 (C6′), 100.48 (C10), 95.92 (C6), 94 (C8), 80.88 (C2), 66.44 (C3), 26.53 (C4). *m*/*z* value in QDa 1 = 290 [M˙^+^] found in R at RT 1.81 min and *m*/*z* value in QDa 1 = 291 [M+H]^+^ found in AP at RT 1.86 min.

#### 2.7.8. 1-*O*-methyl-*epi*-inositol (**8**)

^1^H NMR spectral data (600 MHz, D_2_O), δ: 4.00 (1H, *m*, H2), 3.96 (1H, *m*, H5), 3.92 (1H, *m*, H3), 3.77 (1H, *m*, H4), 3.62 (1H, *m*, H6), 3.43 (1H, *m*, H1), 3.37 (3H, *s*, H7). ^13^C NMR, δ: 84.77 (C1), 75.22 (C4), 72.72 (C3, C5), 71.29 (C2), 66.39 (C6), 60.41 (C7).

### 2.8. TXRF Analysis

Samples were processed in the form of suspensions. Approximately 10 mg of powder samples were suspended in nearly 1000 mg of ultrapure de-ionized water obtained from a Milli-Q purifier system (Millipore DirectQ-5 TM, Millipore S.A.S., 67120, Molsheim, France). The suspensions underwent homogenization using a vortex shaker for 1 min at 2500 rpm. A 10 µL drop of an internal standard solution containing gallium (Ga) was placed at the center of a siliconized quartz sample carrier. This internal standard served as a reference element for quantitative analysis. To create a hydrophobic surface, a silicon solution in isopropanol (Serva Electrophoresis, Heidelberg, Germany) was applied to all quartz glass sample carriers. The sample carriers were then dried on a hot plate at 50 °C under the laminar hood. Subsequently, 10 μL of the prepared sample suspension was deposited onto the previously dried residue on the quartz glass sample carrier and re-dried. Two sample carriers were prepared for each sample, and each set was irradiated for 600 s live time.

TXRF analysis of prepared samples was performed using a commercial benchtop TXRF spectrometer equipped with a Mo low-power X-ray tube (S2 PICOFOX, Bruker AXS Microanalysis GmbH, Berlin, Germany). It is equipped with air-cooled low-power X-ray tubes operating at 750 μA and 50 kV and a Peltier-cooled silicon drift detector (SDD), and thus, no cooling media and gas consumption are required. Spectra evaluation and calculation of the analyte net peak area were performed using the provided software (Spectra Plus 5.3, Bruker AXS Microanalysis GmbH, Berlin, Germany) linked to the system. For peak integration, the Spectra Plus 5.3 software applies a deconvolution routine that uses measured mono-element profiles for the evaluation of peak areas.

### 2.9. Cell Culture and Treatments

Murine macrophage cells (RAW 264.7) (CLS cell lines service, Germany) were cultured in RPMI 1640, supplemented with 10% fetal bovine serum, 1% L-glutamine, 100 U/mL penicillin, and 100 μg/mL streptomycin, in an incubator at 37 °C and 5% CO_2_.

Human neuroblastoma cells (SH-SY5Y) were cultured in DMEM and Ham’s F12 medium 1:1, supplemented with 10% fetal bovine serum, 0.5% L-glutamine, 100 U/mL penicillin and 100 μg/mL streptomycin, in an incubator at 37 °C and 5% CO_2_.

For the treatments, aerial parts (AP) and roots (R) extracts were solubilized in DMSO and diluted in PBS to obtain an initial concentration of 1 mg/mL. The concentrations used for the treatments were then prepared by diluting the extracts in cell culture medium.

To test the biocompatibility of the extracts, RAW 264.7 cells were seeded at a density of 2.4 × 10^3^ cells/mL in 96-well plates. Cells were treated for 48h with AP and R extracts at concentrations of 100 μg/mL, 50 μg/mL, 25 μg/mL, 6.25 μg/mL, 3.12 μg/mL, and 1.56 μg/mL. Untreated cells were used as control. Regarding the SH-SY5Y cell line, the seeding density used was 2 × 10^4^ cells/well in 96-well plates. SH-SY5Y cells were treated for 48h with AP and R extracts using the following dosages: 100 μg/mL, 50 μg/mL, 25 μg/mL, 6.25 μg/mL, 3.12 μg/mL, and 1.56 μg/mL. Untreated cells were used as control.

The extract’s anti-inflammatory activity was tested by treating RAW 264.7 cells with a pro-inflammatory stimulus elicited by LPS for 48 h. Briefly, LPS 500 ng/mL was administered in the presence or absence of AP and R extracts at concentrations between 1.56 μg/mL and 25 μg/mL.

To determine AP and R antioxidant activity, SH-SY5Y cells were pre-treated for 24 h with the extracts at different dosages between 1.56 μg/mL and 6.25 μg/mL. The day after, cells were challenged with 0.2 mM H_2_O_2_ for 24 h.

### 2.10. Cell Viability

After cellular treatments, cell viability was assessed by incubating cells with 500 mg/mL of MTT (3-[4,5-dimethylthiazol-2-yl]-2,5-diphenyl-tetrazolium bromide) for 90 min at 37 °C. After removing the supernatant, cells were lysed with DMSO. The absorbance was measured at 570 nm using an EnSight Multi-mode Plate Reader (PerkinElmer, Waltham, MA, USA). Data were expressed as a percentage of cell viability over the control group. The results were expressed as mean ± SEM.

### 2.11. Catalase Activity

To test catalase activity, SH-SY5Y cells were plated at a density of 2 × 10^5^ cells/well in 24-well plates and treated with AP and R extracts at the most protective dosage. After 48h of incubation, cells were collected and lysate to obtain a total protein extract. Briefly, cells were harvested in 100 μL of lysis buffer containing 1M Tris-HCl pH 7.6, 5N sodium chloride (NaCl), NP-40, and protease and phosphatase inhibitors. Samples were sonicated, and the total protein content was determined using the Bradford assay.

Catalase activity was measured by monitoring the decomposition of H_2_O_2_, according to Shangari and O’Brien [23]. Specifically, 5 μL of total protein extracts were incubated with the substrate (65 µM hydrogen peroxide in 6.0 mM PBS buffer pH 7.4) at 37 °C for 60 s. The enzymatic reaction was stopped by adding 32.4 mM ammonium molybdate, and spectrophotometrically measured at 405 nm. The results were extrapolated by a standard curve (ranging from 12 U/mL to 0.5 U/mL) performed with purified Catalase enzyme (20100 U). Data were finally normalized on the total protein content, and the results were expressed as U/mg.

### 2.12. Mitochondrial Content

SH-SY5Y cells, seeded at a density of 2 × 10^5^ cells/well, were pre-treated for 24 h with AP and R extracts at the most protective dosage and then challenged with 0.2 mM H_2_O_2_ for an additional 24 h. The day after, cells were washed twice with PBS and incubated with MitoTracker™ Green FM 100 nM for 45 min at 37 °C 5% CO_2_. Subsequently, cells were washed again with PBS and then fixed with 3.7% formaldehyde in PBS for 15 min at RT. The observation was performed with the LSM880 Zeiss confocal laser microscope (Carl Zeiss S.p.A., Milan, Italy). Analysis was performed with an excitation wavelength of 490 nm and an emission wavelength of 516 nm. Fluorescence quantification was conducted using Fiji software, an ImageJ2-based program (https://imagej.net/software/fiji/ (accessed on 28 May 2024)).

### 2.13. ATP Production

ATP production in SH-SY5Y cells was analyzed using a Seahorse XFe24 Extracellular Flux Analyzer (Agilent, Santa Clara, CA, USA). SH-SY5Y cells were seeded at a density of 5 × 10^4^ cells/well in Seahorse XFe24 culture plates, previously coated with poly-L-lysine 2 μg/mL, and treated with AP and R 6.25 μg/mL for 48h. Agilent Seahorse XF base medium DMEM (pH 7.4), added with 1 mM sodium pyruvate, 5 mM Glucose, 2 mM glutamine, 5 mM HEPES, was used. At the end of the treatment, cells were incubated at 37 °C in a non-CO_2_ incubator for 1 h before running the assay. To determine mitochondrial ATP production, the ATP synthase inhibitor oligomycin 1.25 µM and a mixture of the respiratory complex I inhibitor rotenone and the complex III inhibitor antimycin A were sequentially injected. Basal respiration was measured in the absence of inhibitors. At the end of each experiment, cells were lysed, and their total protein content was used for the normalization. Protein quantification was assessed, as reported by Tonello et al. [24], by incubating samples in the dark for 15 min with a fluorescent labeling reagent (Qubit protein assay organic dye), and then the fluorescence signal derived from the stained protein was acquired with the Qubit fluorometer (Thermo Fisher Scientific, Waltham, MA, USA).

### 2.14. Quantitative Real-Time PCR

The most promising dosage of the extracts able to protect cells against the LPS stimulus was employed to treat the cells and assess the expression of pro-inflammatory genes such as interleukin-1β (IL-1β) and interleukin-6 (IL-6). Specifically, RAW 264.7 cells were treated with AP and R extracts in the presence or absence of LPS for 6 h. Then, total RNA was extracted from 4 × 10^4^ cells/mL following the TRIzol reagent protocol. Two micrograms of total mRNA were then reverse-transcribed. The reaction mix was composed of oligo (dT) primers 5 mM, 10 U/mL M-MLV reverse transcriptase, 1 mM dNTPs, 1 U/mL RNase inhibitor, and 1X RT buffer. The reaction was conducted at 70 °C for 10 min, followed by a 2 min step at 4 °C and a 60 min step at 37 °C. Real-time PCR was performed using the ViiA7 Real-Time PCR Detection System (Applied Biosystems, Foster City, CA, USA). The reaction mix contained 6 µL of SYBR Green Master Mix, 6 pmol of each forward and reverse primer, and 2 µL of diluted cDNA. It was incubated at 95 °C for 10 min, followed by 40 cycles at 95 °C for 15 s and 60 °C for 60 s. For each gene, samples were plated in triplicate. Primers sequences were IL-1β (F: CTTCAGGCAGGCAGTATC, R: TAATGGGAACGTCACACACC), IL-6 (F: CCTACCCCAATTTCCAATGCT, R: TATTTTCTGACCACAGTGAGGAAT), β-actin (F: AGCCATGTACGTAGCCATCC, R: TCTCAGCTGTGGTGGTGAA). β-actin was used as a calibrator. Relative quantification was performed using the comparative Ct method. Data were presented as the fold change in target gene expression and expressed as mean ± SEM.

### 2.15. Statistical Analysis

The results are presented as mean ± standard error mean (SEM) of three independent replicates. Data were analyzed using a one-way ANOVA test, followed by Dunnett’s test for multiple comparisons. A *p*-value < 0.05 was considered significant. Statistical analyses were performed using GraphPad Prism 9.0.0 (GraphPad Prism Software, San Diego, CA, USA).

## 3. Results

### 3.1. Anti-Inflammatory Potential of C. monspeliensis Extracts

**Figure 2:**
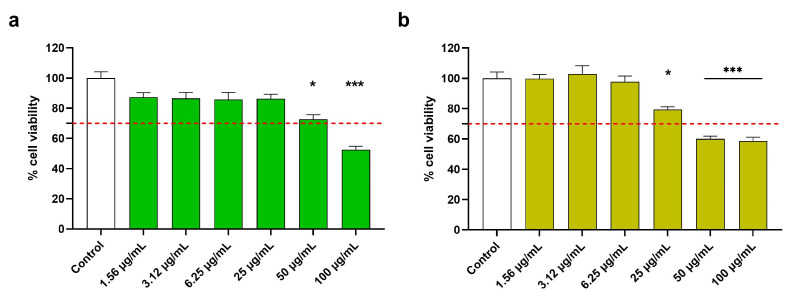
Effect of *C. monspeliensis* aerial parts (**a**) and roots (**b**) extracts on RAW 264.7 cell line viability. Cells were treated with the extracts at concentrations ranging from 100 µg/mL to 1.56 µg/mL for 48 h. Cell viability was assessed with an MTT assay. Data are presented as a percentage of cell viability compared to untreated cells, which are used as a control. The red dotted line indicates the percentage of cell viability under which the treatments were considered cytotoxic. Data are shown as mean ± SEM of three replicates: *** *p* < 0.001, * *p* < 0.05 vs. control group.

Firstly, the cytocompatibility of aerial parts (AP) and roots (R) extracts was investigated on RAW 264.7 by performing an MTT assay. Figure 2 depicts the percentage of viable cells after a 48 h treatment. Specifically, cells were treated with *C. monspeliensis* AP and R extracts at concentrations ranging from 100 µg/mL to 1.56 µg/mL for 48h. Treatments that reduced cell viability by more than 70% were considered cytotoxic. The results showed that the AP extract significantly reduced cell viability at concentrations above 50 µg/mL (EC_50_ 59.4 µg/mL) (Figure 2a), while the R extract showed toxicity from 25 µg/mL (EC_50_ 24.88 µg/mL) (Figure 2b).

**Figure 3:**
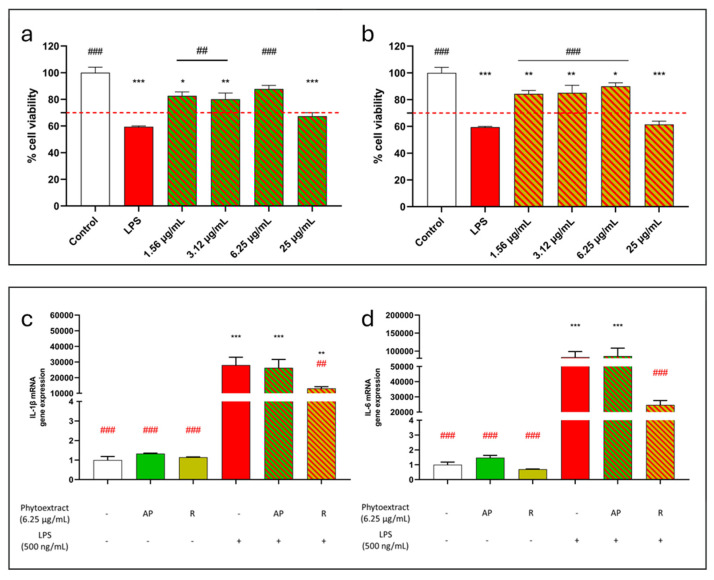
(**a**,**b**) Protective effect of *C. monspeliensis* aerial parts (**a**) and roots (**b**) extracts on RAW 264.7 cells subjected to LPS-induced inflammation. Cells were co-treated with the extracts at concentrations ranging from 25 µg/mL to 1.56 µg/mL and LPS (500 ng/mL) for 48 h. Cell viability was assessed with an MTT assay. Data are presented as a percentage of cell viability compared to untreated cells, which are used as a control. The red dotted line indicates the percentage of cell viability under which the treatments were considered cytotoxic. Data are shown as mean ± SEM of three replicates: *** *p* < 0.001, ** *p* < 0.01, * *p* < 0.05 vs. control group; ### *p* < 0.001, ## *p* < 0.01 vs. LPS. (**c**,**d**) *C. monspeliensis* roots extract reduces the expression of the inflammatory cytokines IL-1β (**c**) and IL-6 (**d**) in the presence of LPS. RAW 264.7 cells were co-treated with *C. monspeliensis* aerial parts (AP) and roots (R) extracts at a concentration of 6.25 µg/mL and LPS (500 ng/mL) for 6 h. Cells were then processed to measure IL-1β and IL-6 mRNA levels by real-time PCR. β-actin was used to normalize the results. Data are shown as mean ± SEM; *** *p* < 0.001, ** *p* < 0.01 vs. control group; ### *p* < 0.001, ## *p* < 0.01 vs. LPS.

In order to test the anti-inflammatory action of *C. monspeliensis*, cells were co-treated with the AP and R extracts at concentrations that did not reduce cell viability by more than 70% (1.56–25 µg/mL). LPS (500 ng/mL) [25,26] was used as an inflammatory stimulus for 48h. According to the results shown in Figure 3, the LPS treatment reduced cell viability by 40%. The treatment with the AP extract in the presence of LPS significantly reduced cytotoxicity at concentrations between 1.56 and 6.25 µg/mL. However, the highest dose used (25 µg/mL) failed to protect against the LPS stimulus, possibly due to the dose-dependent cytotoxicity of the extract previously found (Figure 3a). Likewise, also in the co-treatment with R extract and LPS, the extract showed a protective effect against the LPS inflammatory stimulus at dosages between 1.56 and 6.25 µg/mL, while cell mortality increased at 25 µg/mL (Figure 3b).

To further investigate the anti-inflammatory action of *C. monspeliensis* extracts, cells were treated with the AP and R extracts and LPS for 6 h. Based on the results concerning the protection against the LPS stimulus, the extracts were used at a concentration of 6.25 µg/mL, which was the highest dose that resulted to protect against LPS. Subsequently, the mRNA gene expression of two pro-inflammatory cytokines, IL-1β and IL-6, was determined. Interestingly, only the R extract was able to significantly reduce both IL-1β (Figure 3c) and IL-6 (Figure 3d) mRNA gene expression in the presence of LPS. Contrarily, the treatment with the AP extract did not counteract the LPS action for any of the two cytokines (Figure 3c,d).

### 3.2. Anti-Oxidative Activity of C. monspeliensis Extracts

To assess the anti-oxidative potential of *C. monspeliensis* extracts, SH-SY5Y cells have been selected due to their heightened susceptibility to oxidative damage. This characteristic renders them particularly useful in exploring the efficacy of antioxidant compounds in mitigating oxidative stress-induced cellular damage.

**Figure 4:**
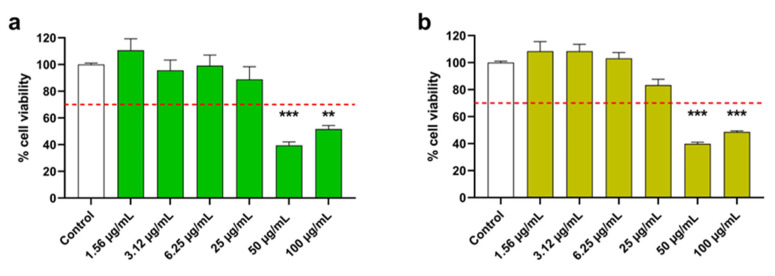
Effect of *C. monspeliensis* aerial parts (**a**) and roots (**b**) extracts on SH-SY5Y cell line viability. Cells were treated with the extracts at concentrations ranging from 100 µg/mL to 1.56 µg/mL for 48 h. Cell viability was assessed with MTT assay. Data are presented as percentage of cell viability compared to untreated cells used as a control. The red dotted line indicates the percentage of cell viability under which the treatments were considered cytotoxic. Data are shown as mean ± SEM of three replicates: *** *p* < 0.001, ** *p* < 0.01 vs. control group.

Initially, the cytocompatibility of *C. monspeliensis* AP and R extracts with SH-SY5Y cells was assessed before examining their antioxidant properties. Also, in this case, cells were treated with *C. monspeliensis* AP and R extracts at concentrations ranging from 100 µg/mL to 1.56 µg/mL for 48 h. Comparable results were observed to those obtained with RAW 264.7 cells; thus, dosages from 6.25 µg/mL to 1.56 µg/mL were further tested (Figure 4).

**Figure 5:**
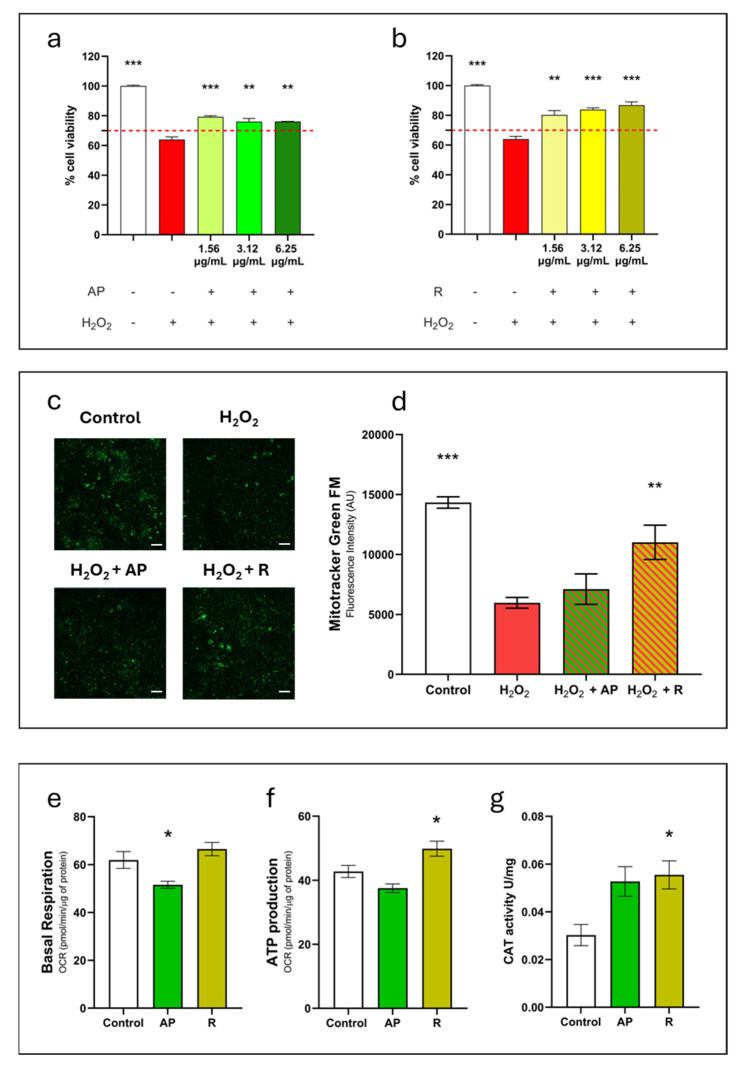
(**a**,**b**) Protective effect of *C. monspeliensis* aerial parts (**a**) and root (**b**) extracts on SH-SY5Y cells subjected to the H_2_O_2_ pro-oxidant stimulus; the red dotted line indicates the percentage of cell viability under which the treatments were considered cytotoxic. Data are shown as mean ± SEM of three replicates: *** *p* < 0.001, ** *p* < 0.01 vs. H_2_O_2_ group. (**c**,**d**) Effect of *C. monspeliensis* aerial parts and roots extracts on mitochondrial functionality. Cells were pre-treated with the extracts at 6.25 µg/mL; H_2_O_2_ was added after 24 h. Mitotracker Green representative figures are shown in (**c**), while in (**d**), the normalized fluorescence intensity graph is shown. Images have been acquired with 10× magnification, white scale bar 50µm. Data are shown as mean ± SEM of three replicates: *** *p* < 0.001, ** *p* < 0.01 vs. H_2_O_2_ group. (**e**,**f**) Graphs relative to basal respiration and ATP production. Cells were treated with *C. monspeliensis* aerial parts and roots extracts at 6.25 µg/mL; cells were evaluated by using the Seahorse XF96e Extracellular Flux Analyzer; (**g**) cellular Catalase enzymatic activity in the presence or absence of AP and R extracts (6.25 µg/mL). Results obtained are normalized on total protein content and expressed as unit/mg of protein. Data are shown as mean ± SEM of three replicates; *p*-value is * *p* < 0.05 vs. control group.

To induce cellular damage, H_2_O_2_ 0.2 mM was utilized as an oxidative stimulus. As depicted in Figure 5a,b, H_2_O_2_ 24 h treatment was able to reduce cell viability by nearly 40%. Cellular pre-treatment with both *C. monspeliensis* AP and R at 6.25 µg/mL was found able to significantly enhance by nearly 20% the cell survival rate after H_2_O_2_ insult, with R (*p* < 0.001) extract outperforming AP (*p* < 0.01).

To explore the potential impact of *C. monspeliensis* extracts on mitochondrial functionality, cells were treated with AP and R at 6.25 µg/mL and subjected to H_2_O_2_ exposure. Mitochondria were then assessed using the MitoTracker fluorescent dye, which selectively stains mitochondria in live cells. Interestingly, we found that pre-treatment with *C. monspeliensis* R extract showed significant increases in MitoTracker Green fluorescent emission when compared to the H_2_O_2_ signal. These data suggest that R extract can promote mitochondria survival when cells are exposed to oxidative stress. On the contrary, no difference in terms of fluorescent signal was observed for the AP extract (Figure 5c,d).

Moreover, it was observed that cells treated with R extract (6.25 µg/mL) exhibited a significantly higher production of ATP compared to untreated cells, suggesting that R extract can exert a positive impact on the cell energy metabolism, improving mitochondria functionality (Figure 5e,f).

To further explore the anti-oxidative properties of AP and R, we investigated the enzymatic activity of Catalase, a crucial antioxidant enzyme that catalyzes the decomposition of H_2_O_2_ into water and molecular oxygen. Data reported in Figure 5g showed that cells treated with R extract (6.25 µg/mL) exhibited a higher catalase enzyme activity when compared to untreated cells. Conversely, cells treated with AP extract (6.25 µg/mL) exhibited higher catalase levels, although the difference was not statistically significant (Figure 5g).

### 3.3. Phytochemical Analysis

A first overview of the phytochemical composition of AP and R extracts was acquired by ^1^H NMR profiling (Figure 6), supported by further pre-purification procedures, 2D NMR, and UHPLC-MS experiments on the obtained fractions (Figures S1–S8).

A singlet at δ 3.46 was prominent in both AP and R ^1^H NMR profiles, and it was attributed to the methoxy group of *O*-methyl-cyclitol; indeed, by HSQC, it correlated with a carbon at δ 58.40. This compound was further purified, and its structure was fully elucidated (Figure S8) as 1-*O*-methyl-*epi*-inositol, a stereoisomer of pinitol. According to the semiquantitative NMR analysis, the concentration of this metabolite was 34.5 µg/mg of AP dry weight (DW) and 22.3 µg/mL of R DW.

Among the primary metabolites, sucrose was also present in both extracts, while α-glucose, β-glucose, and formate were detected only in AP.

**Figure 6:**
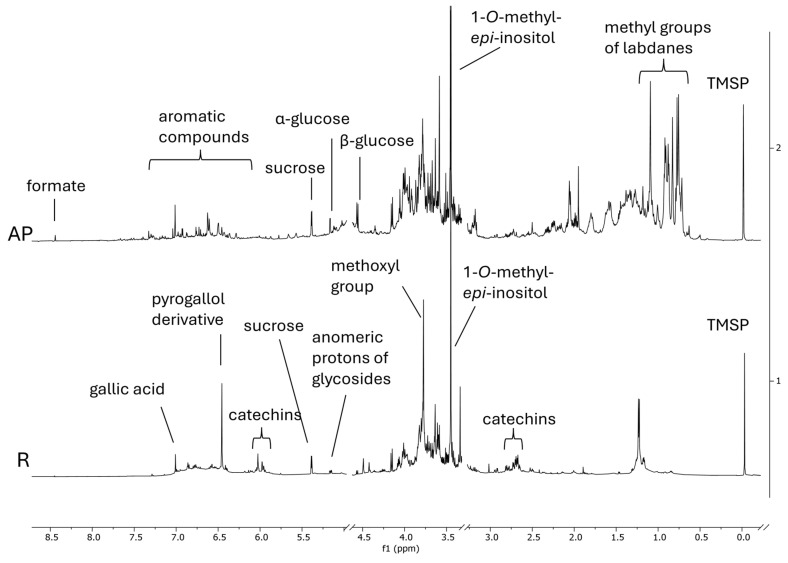
^1^H NMR profiles of *C. monspeliensis* extracts; aerial parts (AP) on the top and roots (R) on the bottom.

Considering the secondary metabolites, the AP and R ^1^H NMR profiles were strongly different. In particular, the AP profile was characterized by several signals in the aliphatic region from δ 0.8 to 2.2. According to the literature, diterpenes are characteristic metabolites of *C. monspeliensis* aerial parts [9], together with methoxylated flavonoids [27,28]. In fact, the AP ^1^H NMR profile showed several singlets diagnostic of labdane terpenes and aromatic signals, potentially ascribable to flavonoids (Figure 6). However, their structure could not be unambiguously characterized only by NMR profiling.

**Figure 7:**
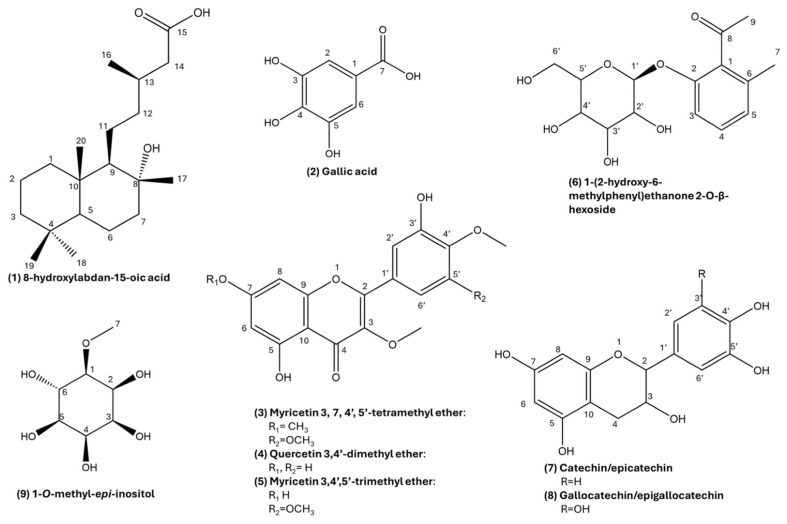
Structures of the most prominent compounds identified.

Through the pre-purification procedure, 8-hydroxylabdan-15-oic acid (**1**) [9] (Figure S2), gallic acid, catechin, methoxylated flavonols, and an acetophenone glycoside (**6**) (Figure 7) were found in AP.

Among the flavonoids found in AP, myricetin 3,7,4′,5′-tetramethyl ether (**3**) has already been reported in *C. monspeliensis* [9,18]. It was easily identified based on the HMBC correlations given by its four methoxyl groups (Figure S3). In particular, the protons of the methoxyl at δ 3.88 correlated with carbon at δ 168, typical of flavonoids A ring. This was, in turn, equally correlated with protons 6 and 8, suggesting that the methoxyl substitution was in position 7. The protons of the other two methoxyl groups (δ 3.83 and 3.78) correlated in HMBC with the carbons at δ 139.2 and 139.4, respectively; these values are diagnostic of carbon 3 and carbon 4′. Indeed, the carbon at δ 139.4 was also correlated with the proton in 6′ (δ 7.22) and the hydroxyl proton in 3′ (δ 9.63) (visible because the sample was prepared in DMSO-*d_6_*), confirming that also position 4′ was methoxy substituted. Considering the splitting pattern of the protons of the B ring (two doublets with J = 2.11 Hz), this aromatic ring was asymmetric; hence, the other methoxyl group (protons at δ 3.87 and carbon at δ 56.40) was positioned on carbon 5′ (δ 153.5). The presence of compound **3** in the total extract was further confirmed by UHPLC-MS analysis, which showed in QDa1 mode a *m*/*z* value 375.24 interpreted as [M+H]^+^.

Compounds **4** and **5** (Figure 7) eluted together in APCl subfraction 8 (Figure S4). The UHPLC-MS analysis of this subfraction (Figure S5) suggested the presence of a quercetin dimethyl ether (*m*/*z* 331.22) and a myricetin trimethyl ether (*m*/*z* 361.23), interpreted both as [M+H]^+^. Actually, the ^1^H NMR spectrum showed signals of protons belonging to two distinct flavonoid A rings (two doublets around δ 6.2 and 6.4. with J = 2.10 Hz), as well as five singlets around δ 3.8, ascribable to the protons of five methoxyl groups. Moreover, the spectrum also showed three signals diagnostic of a catechol moiety and two doublets with both J = 2.10 Hz. The protons of two methoxyl groups correlated with carbons at δ 138.2 and 138.8 (Figure S4), diagnostic of position 3. This information was crucial to assess that both compounds were methoxylated in position 3. One more methoxyl signal correlated with a carbon resonating at δ 139.3, attributed to that in position 4′ of compound **5**, which was identified as myricetin 3,4′,5′-trimethyl ether. Finally, the doublet at δ 6.97 (J = 8.43 Hz) ascribable to proton 5′ of compound **4** (Figure 7) gave HMBC correlation with carbon at δ 148.58, which in turn correlated with the methoxyl protons at δ 3.86 (Figure S4). This allowed us to identify compound **4** as quercetin 3,4′-dimethyl ether.

Compound **6** was found in APEt subfraction 15 (Figure S7). The ^1^H NMR spectrum of this molecule showed three aromatic protons, several signals ascribable to a sugar moiety, and two characteristic singlets resonating at higher fields (δ 2.51 and 2.18).

The HMBC correlations of the proton resonating at δ 2.51 with the carbon at δ 218 and the carbon at δ 131.4 (C1) were diagnostic of an acetophenone structure. Moreover, the carbon at δ 131.43 also correlated in HMBC with the singlet at δ 2.18 (whose carbon established by HSQC correlation resonated at δ 17.91) and was interpreted as a methyl group in position 6. These methyl protons gave, in turn, HMBC correlation also with carbon 6 (δ 135.51) and carbon 5 (δ 125.29) of the aromatic ring.

Moreover, carbon 2 (resonating at δ 152.6) gave HMBC correlation not only with the aromatic protons in 3 (δ 6.98) and in 4 (δ 7.26), but also with a doublet at δ 5.01, which carbon resonates at δ 100.4 (by HSQC). This latter is a typical anomeric proton; hence, the HSQC and HMBC spectra revealed the presence of other signals ascribable to a hexose. By the coupling constant of the anomeric proton (J = 7.7 Hz), it was possible to define that the glycosidic bond was in the *β* orientation. Further purifications are ongoing to determine the stereochemistry of the sugar. Hence, compound **6** was characterized as 1-(2-hydroxy-6-methylphenyl) ethanone 2-*O*-*β*-hexoside. When searching this structure on the online database Reaxys (independently from the sugar stereochemistry), only juniperoside III was found, where the sugar moiety is glucose. This compound was first identified in *Juniperus occidentalis* [29]. Venditti et al. [27] found a compound with a structure similar to **6** in *C. monspeliensis*, with one more hydroxyl substituent.

Other aromatic compounds were found in APEt, particularly gallic acid and catechin (Figure 7 and Figure S6). This latter eluted together with compound **6**. To establish that this compound was catechin and not its isomer epicatechin, the ^1^H NMR signal of proton 2 was diagnostic. In fact, this doublet resonating at δ 4.54 had J = 7.75 Hz, indicating an opposite orientation of the coupling protons in positions 2 and 3.

Gallic acid was also found in R (Figure 6), and it was responsible for the singlet at δ 7.03; its concentration by semiquantitative analysis was 1.7 µg/mL (DW). In addition, the R ^1^H NMR profile showed a particularly abundant derivative of pyrogallol, responsible for the singlet at δ 6.48, correlating with a methoxyl group; however, to fully characterize this compound, other pre-purification procedures are ongoing.

R profile also showed signals ascribable to catechins. The UHPLC-MS analysis revealed the presence of both catechin or its isomer epicatechin (exact mass 290.26 Da), giving 289.11 [M-H]^−^ in QDa 2, and gallocatechin or its isomer epigallocatechin (exact mass 306.267 Da), which gave a *m*/*z* value of 305.11 [M-H]^−^ in QDa 2 (Figure S1).

### 3.4. Total Reflection X-ray Fluorescence (TXRF) Analysis

**Figure 8:**
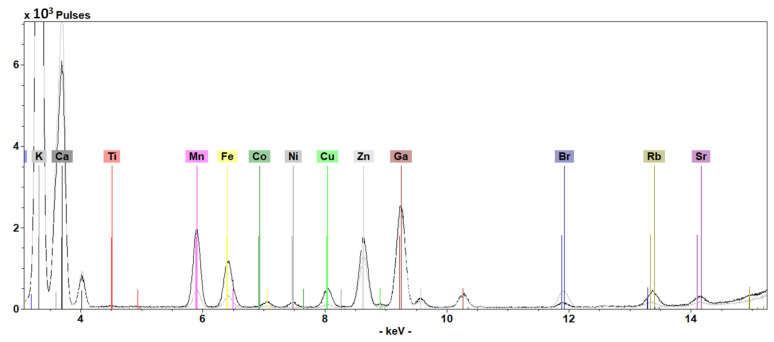
TXRF spectra of *C. monspeliensis* AP (grey line) and R (black line) acquired for 600 s live time.

TXRF spectra of *C. monspeliensis* AP and R extracts are shown in Figure 8.

The inorganic content of *C. monspeliensis* AP and R extracts was determined by means of TXRF analysis. Table 1 shows a clear difference between the two extracts in terms of both qualitative and quantitative determination. The identified elements included macronutrients (K and Ca) and micronutrients (Ti, Mn, Fe, Co, Ni, Cu, Zn, Br, Rb, Sr). The majority of the elements were present in both extracts, except for Ti, which was not detected in the R extract, and Co, not found in the AP extract. From a quantitative point of view, concentrations of K, Ca, and Br were higher in the AP, while Ni, Cu, and Sr were more abundant in the R extract. Minor variations were detected for Mn, Fe, Zn, and Rb. Potentially toxic elements, such as Pb, were not detected in any sample. The differences underscore the selective accumulation of elements within distinct plant tissues [30].

## 4. Discussion

Natural product-derived compounds have always been considered of great importance for drug discovery [31], and ethnobotanical knowledge has often contributed to laying the basis for finding natural molecules and extracts endowed with specific bioactivity [32,33,34].

Due to its large use in Mediterranean traditional medicines [6], the *Cistus* genus has been widely studied, revealing the presence of specialized metabolites endowed with antioxidant, antimicrobial, anti-inflammatory, and neuroprotective properties [3,4,5,6,10,11].

In recent years, interesting data have been reported on *Cistus incanus* L., one of the most well-known *Cistus* species, which grows in the innermost areas of Europe. This plant has shown antioxidant and anti-inflammatory properties [19,35,36].

This work was focused on *Cistus monspeliensis*, from which the aerial parts are used in Sardinian traditional medicine for wound healing [13], a condition with an inflammatory basis [37]. The antioxidant properties of *C. monspeliensis* aerial parts are supported by the literature; however, limited studies have been conducted [5,18,19], and none of them are on cell models.

Hence, this work was addressed to deepen the bioactivity of the ethnobotanically relevant organ (aerial parts: AP), as well as investigate its roots (R), which still remain unexplored both in terms of bioactivity and phytochemistry.

Prior to investigating their anti-inflammatory potential, the biocompatibility of the extracts was assessed for the RAW 264.7 cell line. AP and R did not cause cytotoxicity at concentrations lower than 50 µg/mL and 25 µg/mL, respectively.

When tested for their ability to protect against the LPS inflammatory stimulus, the extracts (AP and R) were active in a concentration range between 1.56 and 6.25 µg/mL.

During inflammation, the pro-inflammatory response is mediated by cytokines, including IL-6, IL-1β, and TNF-α. Their production can be triggered by the recognition of bacterial products, such as LPS and toxins, through the toll-like receptor (TLR) family present in monocytes, macrophages, and endothelial cells [26,38].

Hence, to assess the ability of the extracts to modulate the gene expression of pro-inflammatory cytokines involved in the inflammatory response, the gene expression of IL-6 and IL-1β was investigated. Interestingly, only the treatment with R (at 6.25 µg/mL) was able to reduce the expression of these two pro-inflammatory cytokines in the presence of LPS. In future studies, it will be valuable to assess the anti-inflammatory action of R on specific targets like hyaluronidase and tyrosinase, similar to the approach taken by Graczyk et al. [39].

The data obtained in this study suggest that R has notable antioxidant capabilities, effectively protecting cells from oxidative stress-induced death. These findings suggest that the antioxidant action of R may be mediated by its effects on mitochondria: indeed, the treatment enhanced energy capacity, as evidenced by higher ATP levels in the cells. Additionally, R acted on multiple levels, enhancing the cell’s ability to detoxify an excess H_2_O_2_ through increased catalase enzyme activity. Interestingly, although AP extract protects against oxidative stress-induced cell death, it did not confirm mitochondrial-mediated action. The overall findings let us hypothesize that AP could exert its action through other mechanisms that have not been investigated here.

The metabolite composition of the extracts was first explored by ^1^H NMR profiling, a technique often used to analyze complex mixtures and commonly applied in metabolomics [40,41,42]. Because of the NMR robustness and repeatability, due to the easy sample preparation procedure, the ^1^H NMR fingerprinting is particularly appealing considering data recycling and reuse; for instance, spectral profiles produced in different works can be collected in order to create a database. For this reason, we deposited all the spectra in a data repository (https://doi.org/10.5281/zenodo.11281288).

By ^1^H NMR profiling, it was found that the AP and R have only some primary metabolites in common, such as sucrose and cyclitol, which resulted in 1-*O*-methyl-*epi*-inositol. This latter was one of the most prominent compounds in both AP and R, with a concentration of 34.5 µg/mg (DW) and 22.3 µg/mL (DW), respectively. 1-*O*-methyl-*epi*-inositol is a stereoisomer of the most common pinitol, which was already reported in *C. monspeliensis* [27].

Ciclitols generally act as osmoprotectants in plants, allowing them to survive under water stress due to drought and high salinity [43,44]. They are also endowed with several bioactivities; for instance, pinitol was proven active as antidiabetic, antioxidant, anti-inflammatory, anti-cancer, and chemopreventive [45].

The ^1^H NMR profiling also showed a high amount of labdane terpenes in AP; in fact, *C. monspeliensis* is renowned for its aromatic resin that coats the leaves, constituted mainly of labdanes and clerodanes [27].

Deepening the phytochemical investigation, several methoxy flavonols were found exclusively in AP, while catechins were found in both AP and R. 8-hydroxylabdanoic acid and myricetin 3,7,4′,5′-tetramethyl ether were already detected in Sardinian *C. monspeliensis* ecotypes by Salomè-Abarca et al. [9], while quercetin 3,4′-dimethyl ether and myricetin 3,4′,5′-trimethyl ether are here reported for the first time in a species of *Cistus* genus.

The phytochemical investigation conducted in this work on AP also yielded another compound, which is apparently rare in plants and new to *Cistus*, namely 1-(2-hydroxy-6-methylphenyl) ethenone 2-*O*-*β*-hexoside. No studies on the biological activity of this compound are available.

The high degree of methylation appears to be a characteristic phytochemical signature of the *C. monspeliensis* metabolome; in fact, from 1-*O*-methyl-*epi*-inositol to many aromatic compounds found in this plant (both AP and R), they all bear methyl groups on their structure, either *C*-bonded or *O*-bonded (methoxy).

Although further studies will be necessary to assess which compounds are responsible for the bioactivities measured, it is reasonable to hypothesize that flavonoids, found both in AP and R, might make an important contribution since they are renowned natural antioxidants. In particular, among flavonoids, catechins, which appear more abundant in R than AP by ^1^H NMR profile, might have a prominent role in determining the bioactivities observed [46,47]. Moreover, the pyrogallol moiety was found both on metabolites contained in AP and R. This moiety is renowned for enhancing antioxidant activity [48], and recently, pyrogallol derivatives have also been associated with in vitro antiproliferative activity [49].

Even if the bioactivity of a plant extract is generally associated with its metabolites, the bioactivity and the content of plant secondary metabolites can also be influenced by the presence of essential heavy metals [20].

Plant elements can be subdivided in macronutrients, e.g., Nitrogen (N), Phosphorus (P), Potassium (K), Calcium (Ca), Sulfur (S), and Magnesium (Mg) and micronutrients, e.g., Iron (Fe), Zinc (Zn), Copper (Cu), Boron (B), Manganese (Mn); the former are needed in large amounts, while the latter in traces and are beneficial for plant growth and development [50]. Since plants take essential and non-essential elements from the soil, including potentially toxic heavy metals, their content depends on both the environment and human activities, such as mines, foundries, and agriculture [51,52]. Also, it is established that roots function as a barrier, limiting the transfer of heavy metals to the upper sections of the plant. The accumulation of heavy metals is more pronounced in the roots, gradually decreasing as it moves towards the upper parts (roots > stems > leaves > crops) [52].

It is worth mentioning that the antioxidant activity of some flavonoids, e.g., quercetin, rutin, galangin, complexed with metal ions, was found to be higher than that of free flavonoids; moreover, metal ions coordinated with rutin can potentiate the anti-inflammatory activity [20]. Several studies have analyzed the influence of heavy metals on the metabolomic profile and biological action of plants. Lala showed that the application of Cu nanoparticles at sub-toxic doses increased the whole secondary metabolites content in *Bacopa monnieri* [53]; Slaven Jurić and colleagues applied microparticles containing Ca and Cu ions to augment the secondary metabolites production in *Lactuca sativa* L. [54].

According to Mucha and colleagues [20], the antioxidant activity of metal ions, specifically Al(III), Zn(II), Cu(II), and Fe(II) complexed with flavonoids was higher than that of the single compounds. Complexes of Cu(II), Fe(II), Fe(III), Ni(II), and Zn(II) with rutin also showed better anti-inflammatory activity [20].

Hence, considering the importance of the inorganic composition of a plant, the extract’s elemental content was also determined. Also in this case, AP and R differed from both a qualitative and quantitative point of view. The TXRF analysis revealed the presence of K and Ca (as macronutrients) and Ti, Mn, Fe, Co, Ni, Cu, Zn, Br, Rb, and Sr (as micronutrients). Certain elements were exclusively identified in specific extracts; for instance, Ti was found in the AP, while Co was solely present in the root extract. In addition, K, Ca, and Br were more concentrated in the AP, while Ni, Cu, and Sr had a higher concentration in the R.

In conclusion, it is here shown that the roots of this plant are even more promising as anti-inflammatory than aerial parts, which are traditionally used for this purpose.

None of the extracts were found to accumulate harmful metals. Further studies are ongoing to test single metabolites in bioassays, with the final aim of establishing their contribution to the bioactivity of the raw extracts and to further support the potential use of this plant and its metabolites as active ingredients for anti-inflammatory and antioxidant phytotherapeutic formulations.

## Figures and Tables

**Table 1:** Elemental concentration (mg/kg) of AP and R extracts of *C. monspeliensis*.

Element	AP (mg/kg)	R (mg/kg)
K	9000 ± 2600	1800 ± 550
Ca	1400 ± 400	400 ± 120
Ti	2.1 ± 1.7	-
Mn	38 ± 11	40 ± 13
Fe	34 ± 11	21 ± 7
Co	-	1.5 ± 0.4
Ni	2.3 ± 1.8	5.7 ± 1.6
Cu	5.0 ± 1.5	18 ± 6
Zn	21 ± 6	27 ± 8
Br	8 ± 2	0.9 ± 0.3
Rb	2.7 ± 0.8	2.3 ± 0.8
Sr	0.9 ± 0.3	1.3 ± 0.4
Pb	-	-

## Data Availability

The spectral data are all available in the Zenodo repository (https://doi.org/10.5281/zenodo.11281288). The bioactivity data are available on request from the corresponding author.

## Supplementary Materials

The following supporting information can be downloaded at: https://www.mdpi.com/article/10.3390/antiox13070753/s1, Figure S1: UHPLC-MS analysis of R. Mass spectra in negative ion mode -QDa 2. (A) Mass spectrum at retention time 0.535 min interpreted as gallic acid (exact mass 170.12 Da) which gave *m*/*z* value 169.08 [M-H]^−^. (B) Mass spectrum at retention time 0.760 min interpreted as gallocatechin or epigallocatechin (exact mass 306.267 Da) which gave *m*/*z* value 306.01 [M˙^+^]. (C) Mass spectrum at retention time 1.818 min interpreted as catechin or epicatechin (exact mass 290.26 Da) which gave *m*/*z* value 290.12 [M˙^+^]; Figure S2: NMR-based structural elucidation of 8-hydroxylabdan-15-oic acid (A) Superimposed HSQC and HMBC spectra of APCl subfraction 8 from which the compound was characterized (green dots = HMBC correlations; blue and red = HSQC correlations). (B) Structure of the compound (C) Spectral range between δ 0.60–1.03 highlighting the signals of the methyl groups; Figure S3: Structural elucidation of myricetin 3,7,4′,5′-tetramethyl ether (A) Superimposed HSQC (green dots) and HMBC (red dots) spectra in deuterated DMSO of APEt subfraction 1 from which the compound was characterized. (B) Mass spectra in QDa 1 of the same subfraction showing *m*/*z* 375.24 interpreted as [M+H]^+^ (exact mass of myricetin 3,7,4′,5′-tetramethyl ether 374.10 Da); Figure S4: Structural elucidation of quercetin 3,4′-dimethyl ether (A) and myricetin 3,4′,5′-trimethyl ether (B) Superimposed HSQC and HMBC spectra of APCl subfraction 8 from which the two compounds were characterized (A and B bottom). The blue arrows indicate the HMBC correlations which were crucial to determine the position of methoxyl groups on the molecules; Figure S5: UHPLC-MS analysis of APCl subfraction 8 (A) Chromatogram at 260 nm (B) Mass spectra of peak at RT 5.84 min showing *m*/*z* 331.22 interpreted as [M+H]^+^ (exact mass of quercetin 3,4’-dimethyl ether = 330.0740 Da) (C) Mass spectra of peak at RT 6.045 min showing *m*/*z* 361.23 interpreted as [M+H]^+^ (exact mass of myricetin 3,4’,5’-trimethyl ether = 360.0845 Da); Figure S6: NMR-based structural elucidation of gallic acid and catechin (A) Structures of the two molecules of interest, (B) HMBC spectrum of subfraction 14 of APEt showing the heteronuclear correlations in gallic acid molecule. (C,D) superimposed HSQC and HMBC spectra of APEt subfraction 15 from which catechin was characterized; Figure S7: NMR-based structural elucidation of 1-(2-hydroxy-6-methylphenyl) ethanone 2-*O-β*-hexoside (A) Molecular structure (B) Superimposed HSQC and HMBC spectra of APEt subfraction 15 from which the compound was characterized. The extended regions (C,D) show the correlations between aromatic protons and glycosidic protons, respectively; Figure S8: NMR-based structural elucidation of 1-*O*-methyl-*epi*-inositol in D_2_O (A) Molecular structure, (B) HMBC spectrum of RW subfraction 20 from which the compound was characterized, (C) 2D-NOESY, (D) COSY (the diagonal was suppressed).
