# Supplementary material for: Exploring the Anti-Inflammatory and Antioxidant Potential, Metabolite Composition and Inorganic Profile of Cistus monspeliensis L. Aerial Parts and Roots
Source: Antioxidants (Basel). 2024 Jun 21;13(7):753. doi: 10.3390/antiox13070753 (PMC11273841; doi:10.3390/antiox13070753)
Supplement: Supplementary file 1 [file antioxidants-13-00753-s001.zip › antioxidants-3056473-supplementary material corrected.pdf]

antioxidants

MDPI

Supplementary material

Article

Exploring the Anti-Inflammatory and Antioxidant Potential, Metabolite Composition and Inorganic Profile of *Cistus monspeliensis* L. Aerial Parts and Roots

Eileen Mac Sweeney [1,†], Ilaria Chiocchio [2,†], Manuela Mandrone [2,*], Cinzia Sanna [3], Fabjola Bilo [4], Giuseppina Maccarinelli [1], Vlad Sebastian Popescu [1], Mariachiara Pucci [1], Stefania Morandini [1], Maurizio Memo [1], Daniela Letizia Uberti [1], Laura Borgese [4], Simona Trincia [2], Ferruccio Poli [2], Andrea Mastinu [1,*] and Giulia Abate [1]

[1] Department of Molecular and Translational Medicine, Division of Pharmacology, University of Brescia, 25123 Brescia, Italy; e.macsweeney@studenti.unibs.it (E.M.S.); giuseppina.maccarinelli@unibs.it (G.M.); vlad.popescu@unibs.it (V.S.P.); mariachiara.pucci@unibs.it (M.P.); stefania.morandini@unibs.it (S.M.); maurizio.memo@unibs.it (M.M.); daniela.uberti@unibs.it (D.I.U.); giulia.abate@unibs.it (G.A.)

[2] Department of Pharmacy and Biotechnology (FaBit), Alma Mater Studiorum, University of Bologna, Via Irnerio 42, 40126 Bologna, Italy; ilaria.chiocchio2@unibo.it (I.C.); simona.trincia2@unibo.it (S.T.); ferruccio.poli@unibo.it (F.P.)

[3] Department of Life and Environmental Sciences, University of Cagliari, Via S. Ignazio da Laconi 13, 09123 Cagliari, Italy; cinziasanna@unica.it

[4] Department of Mechanical and Industrial Engineering, University of Brescia, Via Branze 38, 25123 Brescia, Italy; fabjola.bilo@unibs.it (F.B.); laura.borgese@unibs.it (L.B.)

* Correspondence: manuela.mandrone2@unibo.it (M.M.); andrea.mastinu@unibs.it (A.M.)

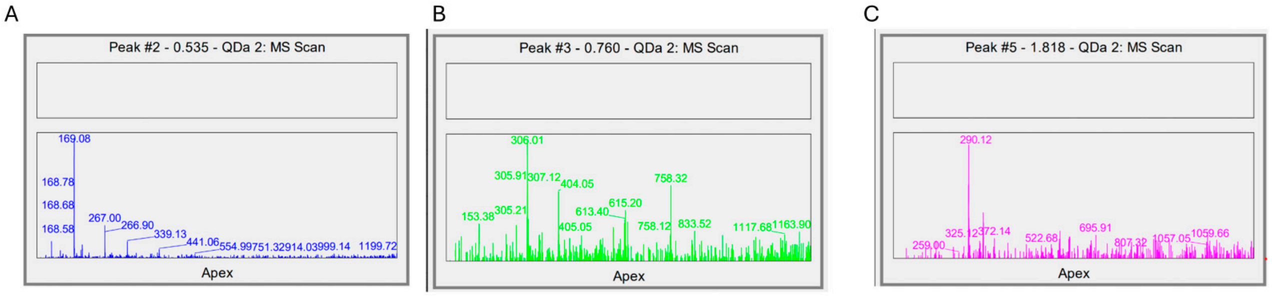

Figure S1. UHPLC-MS analysis of R. Mass spectra in negative ion mode -QDa 2- A) Mass spectrum at retention time 0.535 min interpreted as gallic acid (exact mass 170.12 Da) which gave m/z value 169.08 [M-H]. **B)** Mass spectrum at retention time 0.760 min interpreted as gallocatechin or epigallocatechin (exact mass 306.267 Da) which gave m/z value 306.01 [M $^{\cdot+}$].**C)** Mass spectrum at retention time 1.818 min interpreted as catechin or epicatechin (exact mass 290.26 Da) which gave m/z value 290.12 [M $^{\cdot+}$].

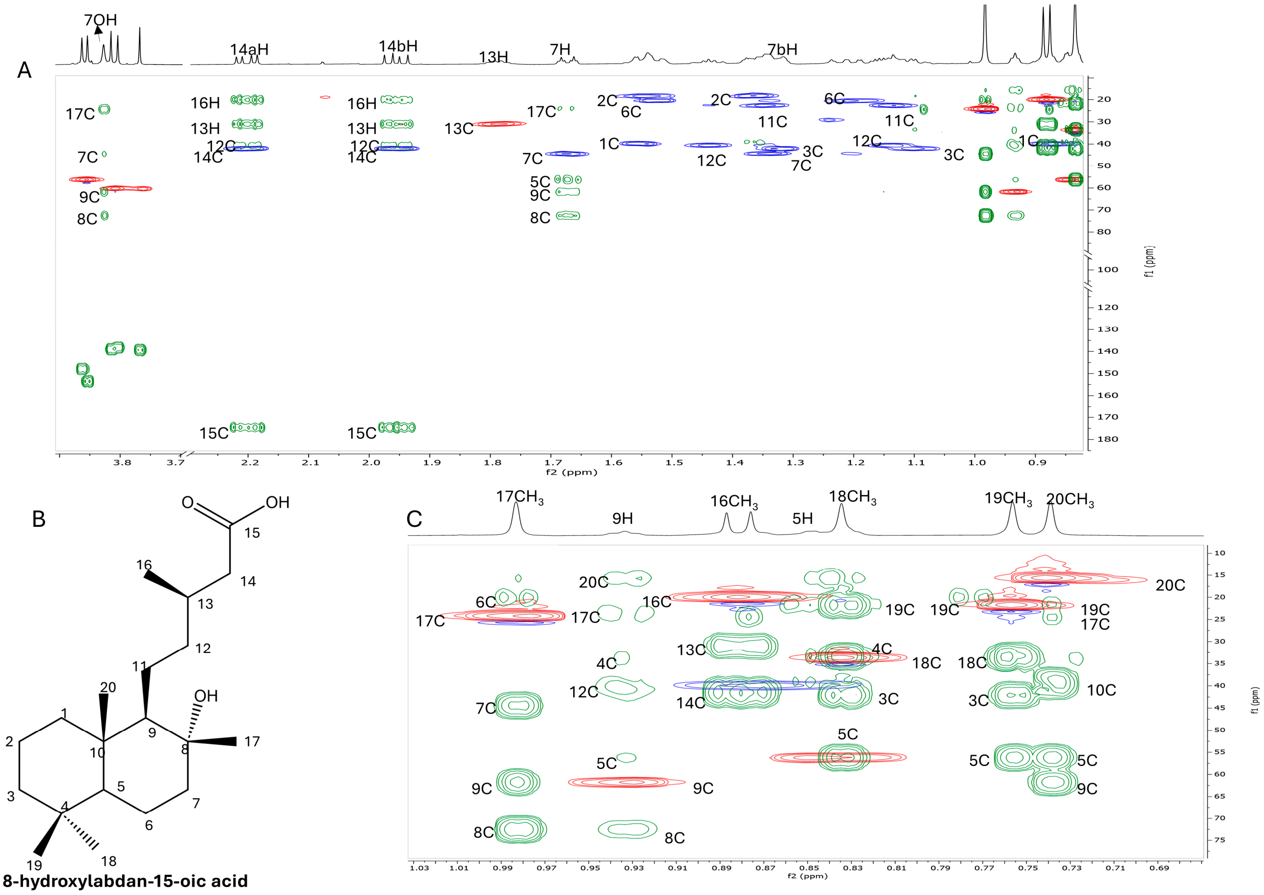

(1) 8-hydroxylabdan-15-oic acid

Figure S2. NMR-based structural elucidation of 8-hydroxylabdan-15-oic acid A) Superimposed HSQC and HMBC spectra of APCl subfraction 8 from which the compound was characterized (green dots = HMBC correlations; bule and red = HSQC correlations). **B)** Structure of the compound **C)** Spectral range between δ 0.60 - 1.03 highlighting the signals of the methyl groups.

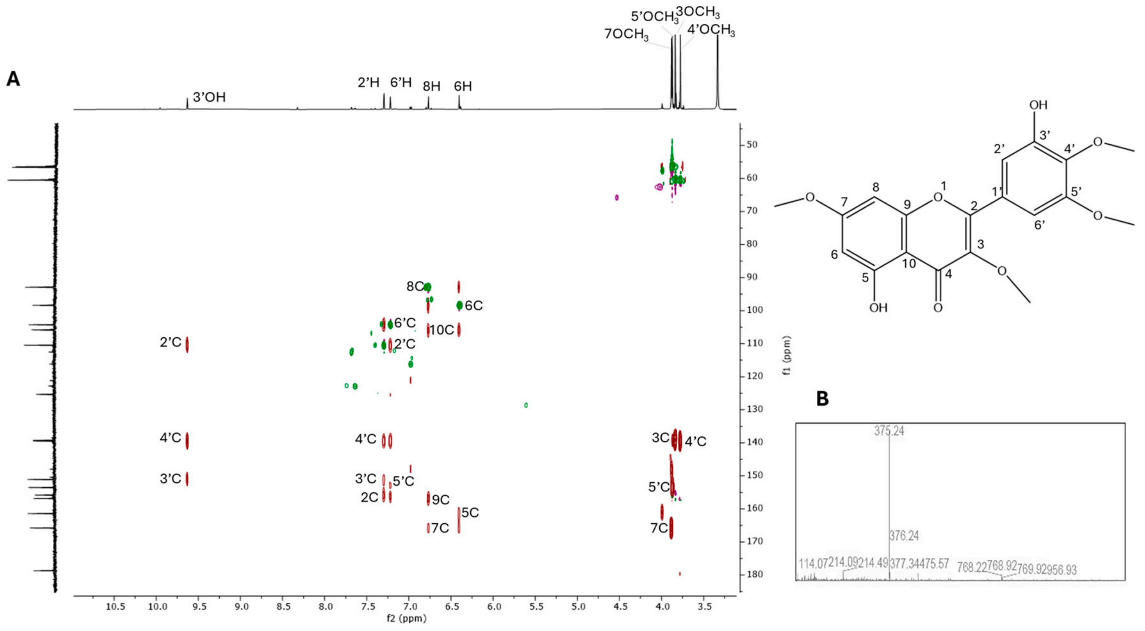

Figure S3. Structural elucidation of myricetin 3,7,4′,5′-tetramethyl ether. A) Superimposed HSQC (green dots) and HMBC (red dots) spectra in deuterated DMSO of APEt subfraction 1 from which the compound was characterized. **B)** Mass spectra in QDa 1 of the same subfraction showing m/z 375.24 interpreted as $[M+H]^+$ (exact mass of myricetin 3,7,4′,5′-tetramethyl ether 374.10 Da).

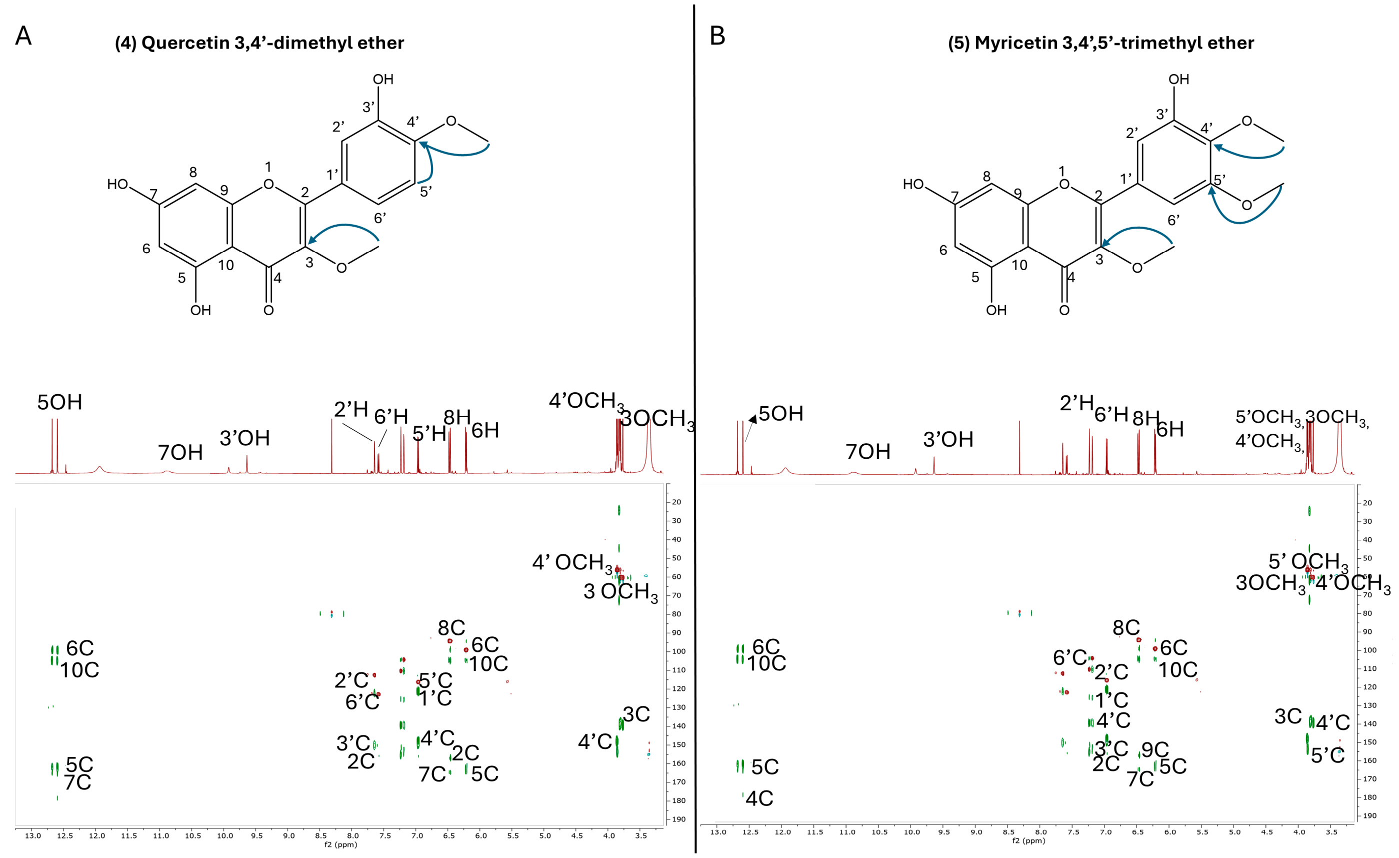

Figure S4. Structural elucidation of quercetin 3,4′-dimethyl ether (A) and myricetin 3,4′,5′-trimethyl ether (B). Superimposed HSQC and HMBC spectra of APCl subfraction 8 from which the two compounds were characterized (**A** and **B** bottom). The blue arrows indicate the HMBC correlations which were crucial to determine the position of methoxyl groups on the molecules.

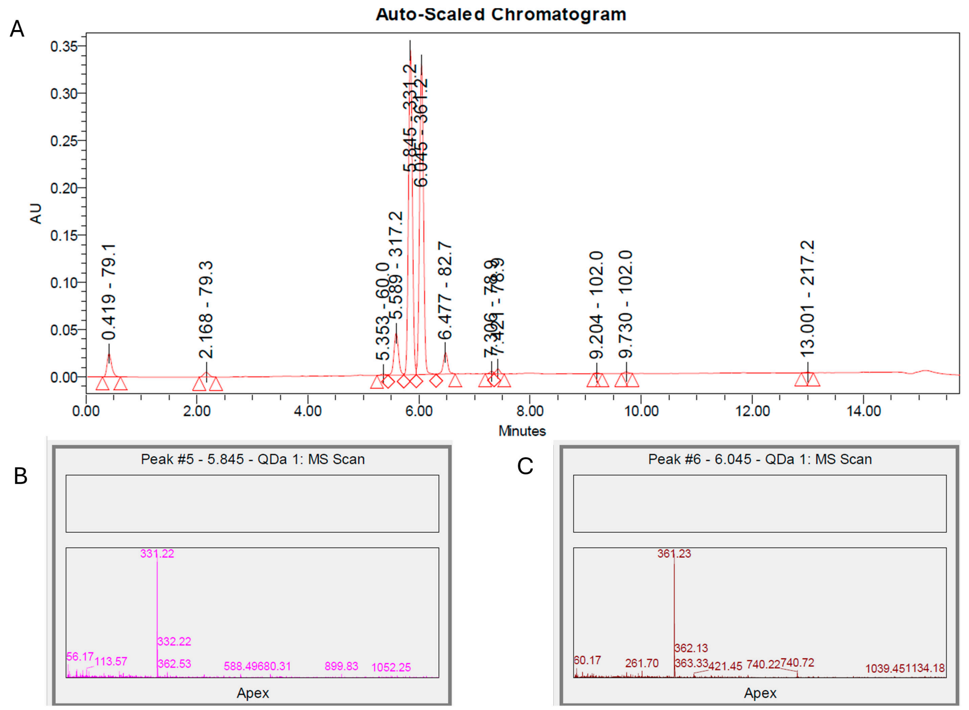

Figure S5. UHPLC-MS analysis of APCl subfraction 8 A) Chromatogram at 260 nm **B)** Mass spectra of peak at RT 5.84 min showing m/z 331.22 interpreted as $[M+H]^+$ (exact mass of quercetin 3,4′-dimethyl ether = 330.0740 Da) **C)** Mass spectra of peak at RT 6.045 min showing m/z 361.23 interpreted as $[M+H]^+$ (exact mass of myricetin 3,4′,5′-trimethyl ether = 360.0845 Da).

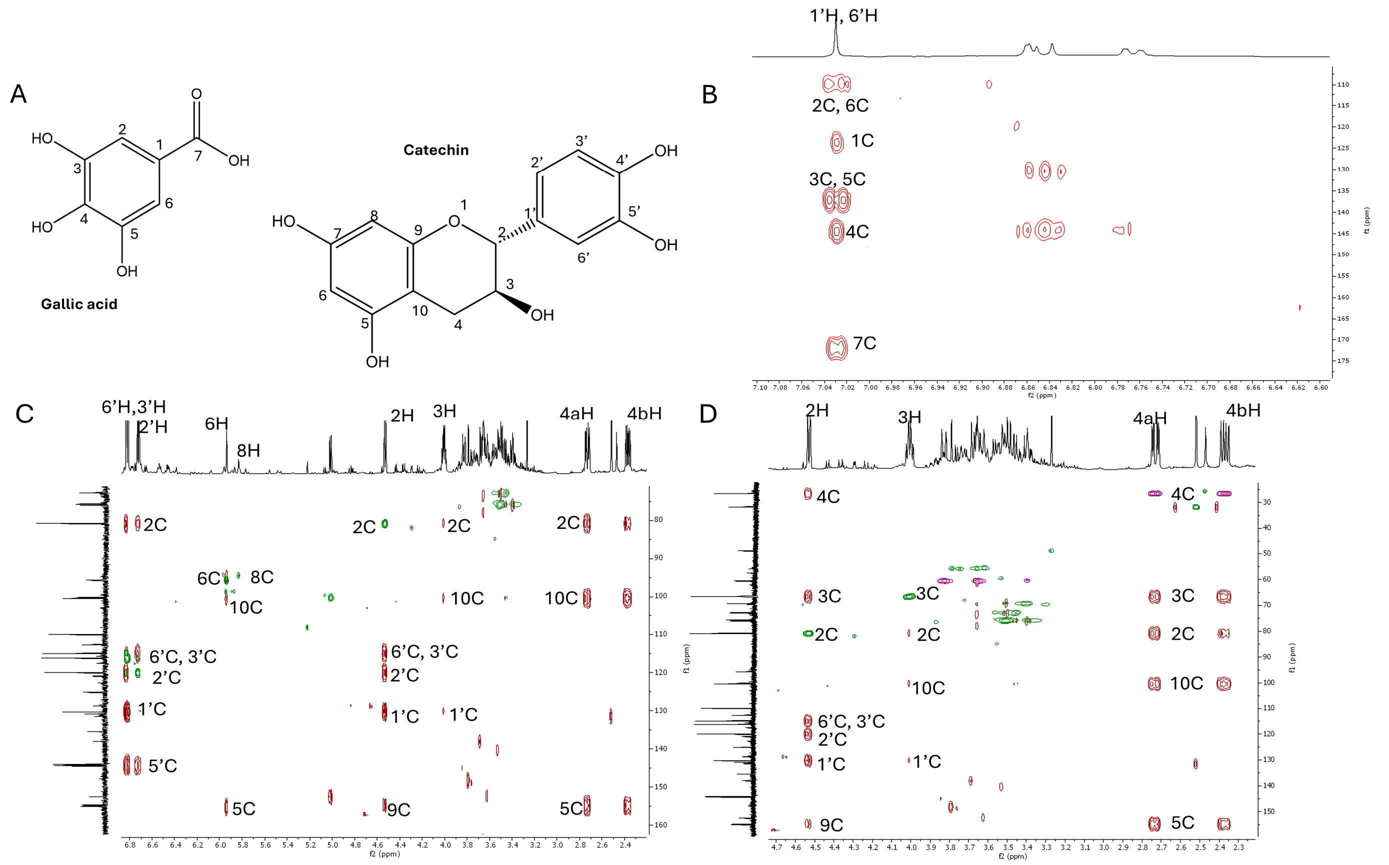

Figure S6. NMR-based structural elucidation of gallic acid and catechin. A) Structures of the two molecules of interest, **B)** HMBC spectrum of subfraction 14 of APEt showing the heteronuclear correlations in gallic acid molecule. **C)** and **D)** superimposed HSQC and HMBC spectra of APEt subfraction 15 from which catechin was characterized.

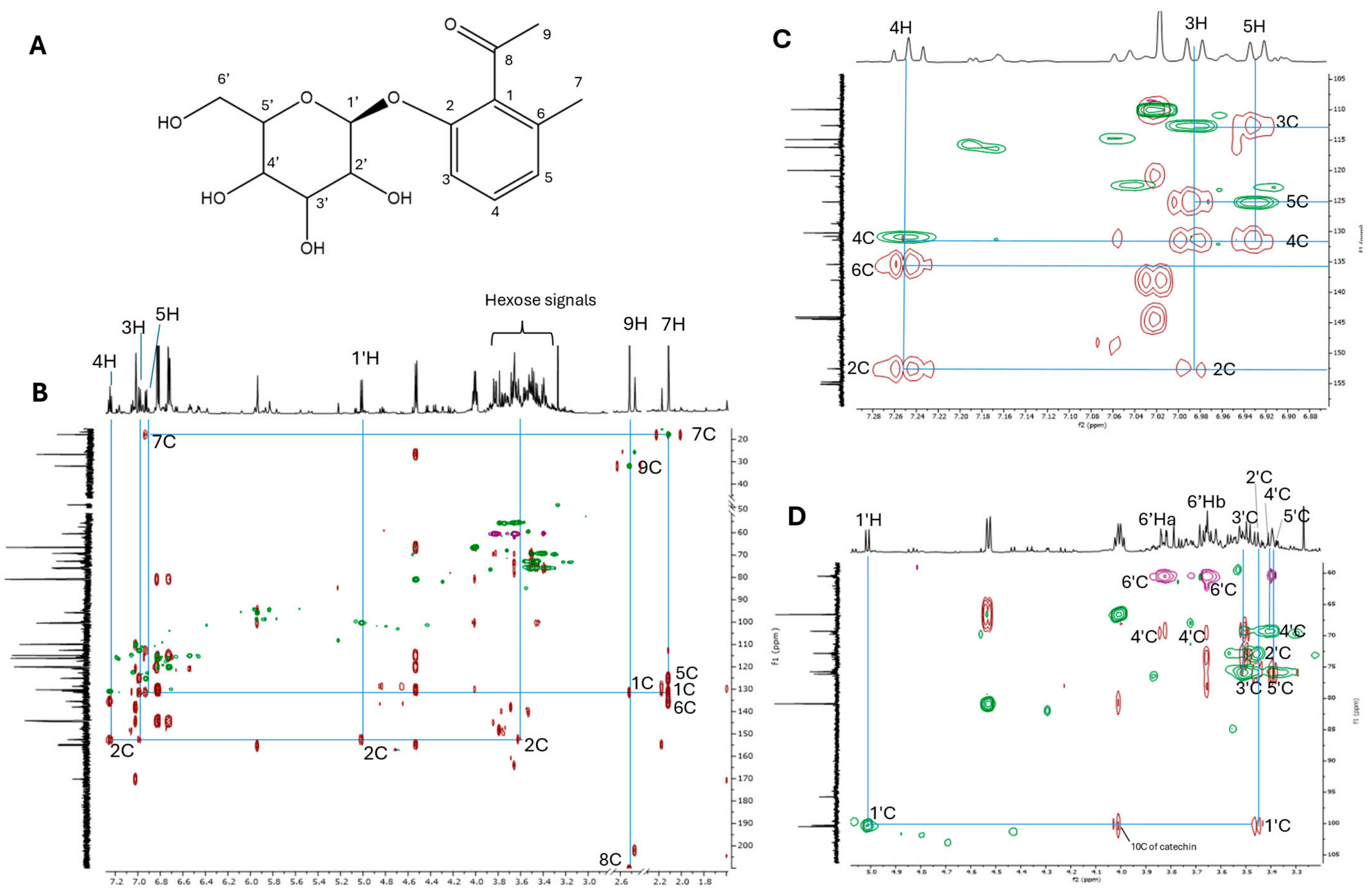

Figure S7. NMR-based structural elucidation of 1-(2-hydroxy-6-methylphenyl)ethanone 2-O-β-hexoside. A) Molecular structure **B)** Superimposed HSQC and HMBC spectra of APEt subfraction 15 from which the compound was characterized. The extended regions **C)** and **D)** show the correlations between aromatic protons and glycosidic protons, respectively.

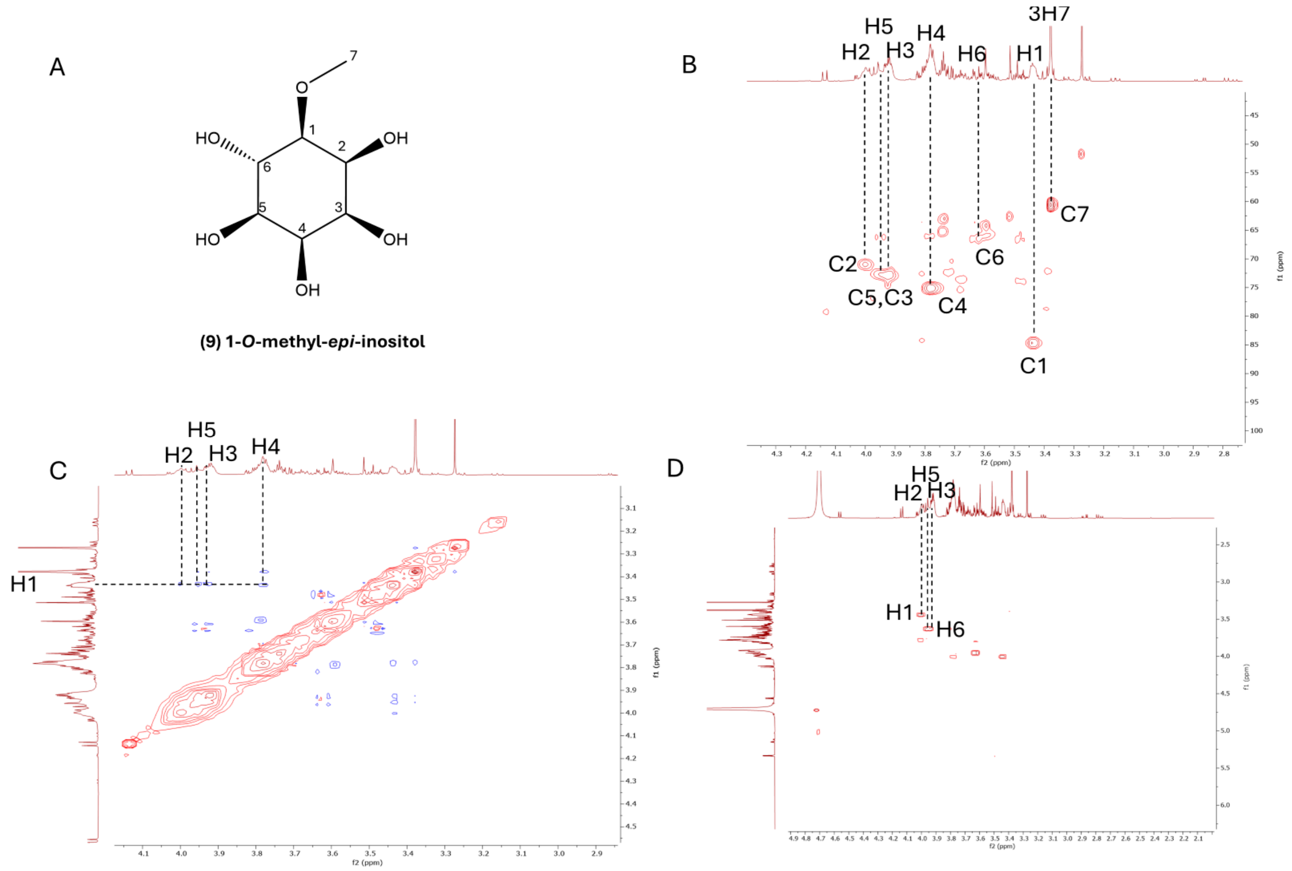

Figure S8. NMR-based structural elucidation of 1-*O*-methyl-*epi*-inositol in D_2O A) Molecular structure, **B)** HMBC spectrum of RW subfraction 20 from which the compound was characterized, **C)** 2D-NOESY, **D)** COSY (the diagonal was suppressed)
